# The predicting roles of carcinoembryonic antigen and its underlying mechanism in the progression of coronavirus disease 2019

**DOI:** 10.1186/s13054-021-03661-y

**Published:** 2021-07-03

**Authors:** Runzhi Huang, Tong Meng, Qiongfang Zha, Kebin Cheng, Xin Zhou, Junhua Zheng, Dingyu Zhang, Ruilin Liu

**Affiliations:** 1 Department of Respiratory and Critical Care Medicine, Tongji Hospital, School of Medicine, Tongji University, Shanghai, 200065 China; 2 Key Laboratory of Spine and Spinal Cord Injury Repair and Regeneration (Tongji University), Ministry of Education, Shanghai, 200065 China; 3 Shanghai General Hospital, 100 Haining Road, Shanghai, 200080 China; 4 Department of Respiratory and Critical Care Medicine, Ren Ji Hospital, School of Medicine, Shanghai Jiao Tong University, 160 Pujian Road, Shanghai, 200127 China; 5 Department of Respiratory and Critical Care Medicine, Shanghai Pulmonary Hospital, Tongji University School of Medicine, Shanghai, 200433 China; 6 Department of Respiratory and Critical Care Medicine, Shanghai General Hospital, 100 Haining Road, Shanghai, 200080 China; 7 Tongji University Cancer Center, Shanghai Tenth People’s Hospital, Tongji University School of Medicine, 301 Yanchang Road, Shanghai, 200072 China; 8 Wuhan Jinyintan Hospital, Wuhan, 430023 China

**Keywords:** Coronavirus disease 2019, Carcinoembryonic antigen, Carcinoembryonic antigen-related cell adhesion molecules, Developing neutrophils, Type II pneumocyte

## Abstract

**Background:**

The coronavirus disease 2019 (COVID-19) has induced a worldwide epidemiological event with a high infectivity and mortality. However, the predicting biomarkers and their potential mechanism in the progression of COVID-19 are not well known.

**Objective:**

The aim of this study is to identify the candidate predictors of COVID-19 and investigate their underlying mechanism.

**Methods:**

The retrospective study was conducted to identify the potential laboratory indicators with prognostic values of COVID-19 disease. Then, the prognostic nomogram was constructed to predict the overall survival of COVID-19 patients. Additionally, the scRNA-seq data of BALF and PBMCs from COVID-19 patients were downloaded to investigate the underlying mechanism of the most important prognostic indicators in lungs and peripherals, respectively.

**Results:**

In total, 304 hospitalized adult COVID-19 patients in Wuhan Jinyintan Hospital were included in the retrospective study. CEA was the only laboratory indicator with significant difference in the univariate (P < 0.001) and multivariate analysis (P = 0.020). The scRNA-seq data of BALF and PBMCs from COVID-19 patients were downloaded to investigate the underlying mechanism of CEA in lungs and peripherals, respectively. The results revealed the potential roles of CEA were significantly distributed in type II pneumocytes of BALF and developing neutrophils of PBMCs, participating in the progression of COVID-19 by regulating the cell–cell communication.

**Conclusion:**

This study identifies the prognostic roles of CEA in COVID-19 patients and implies the potential roles of CEACAM8-CEACAM6 in the progression of COVID-19 by regulating the cell–cell communication of developing neutrophils and type II pneumocyte.

**Supplementary Information:**

The online version contains supplementary material available at 10.1186/s13054-021-03661-y.

## Introduction

In December 2019, the coronavirus disease 2019 (COVID-19) has been out breaking in Wuhan China and rapidly spread throughout the world inducing a worldwide panic [1]. The novel coronavirus was isolated from human airway epithelial cells and was named severe acute respiratory syndrome-related coronavirus 2 (SARS-CoV-2), which is highly infectious and induces a high fatality [2–5]. Nowadays, the underlying pathogenic mechanism of SARS-CoV-2 has been generally explored [6]. Similar to SARS-CoV-1, SARS-CoV-2 uses the receptors of angiotensin converting enzyme II (ACE2) for viral entry process. After receptor binding, the spike (S) protein priming protease, such as cell surface transmembrane serine protease (TMPRSSs) and endosomal cathepsins, works in membrane fusion [2, 7]. However, these proteases are not prognosis predictors of COVID-19 patients, which may be significantly associated with therapeutic decision-making.

Generally, patients’ characteristics, nutritional status, clinical symptoms, comorbidities, inflammatory biomarkers and chest CT images are different in terms of patient outcome; however, whether these factors can serve as prognosis predictors for COVID-19 pneumonia is not clear. Regarding chest CT images, consolidation, emphysema and residual healthy lung parenchyma are regarded as independent predictors in COVID-19 patients [8]. Additionally, high-sensitivity C-reactive protein–albumin ratio (HsCAR) and low prognostic nutritional index (PNI) and the ratio of interleukin (IL)-6 to IL-10 were reported to be related to the prognosis of COVID-19 patients [9, 10]. Moreover, carcinoembryonic antigen (CEA) is a glycoprotein generated in colonic epitheliums in the embryonic period and has been widely used as a biomarker for tumorgenesis and progression. CEA has been also reported to be associated with the prognosis of COVID-19 patients [11, 12]. However, the potential mechanism of their predicting roles is unknown, neither is other candidate predictors.

The aim of this study is to provide novel predictors and their hypothetical mechanism in the infection and progression of COVID-19. In this study, we systematically collected and analyzed clinical information from hospitalized adult patients with COVID-19 pneumonia including demographics, disease, treatment and outcome information to identify all potential prognosis indicators for COVID-19 pneumonia. Based on the identified predictors, the prognostic nomogram was established to guide clinical decision-making. Furthermore, in order to explore the underlying mechanism of candidate biomarkers, single-cell transcriptomics of bronchoalveolar lavage fluid (BALF) from patients with or without COVID-19 were also analyzed with integrated bioinformatics methods. This study will provide novel predictors and their potential mechanism in the infection and progression of COVID-19.

## Materials and methods

### Patient selection and data extraction

This study was approved by the Ethics Committee of Jinyintan Hospital (KY-2020-58.01), followed the Standards for Reporting of Diagnostic Accuracy Studies Statement and Strengthening the Reporting of Observational Studies in Epidemiology (STROBE) [13, 14]. A total of 300 hospitalized adult COVID-19 patients diagnosed by reverse transcription polymerase chain reaction (RT-PCR) in Wuhan Jinyintan Hospital from January 1, 2020, to April 30, 2020, were included in the retrospective study. The exclusion criteria were: (1) patients younger than 18; (2) non-hospitalized patients; (3) patients with follow-up period less than 60 days; (4) patients admitted for another reason than COVID-19-related respiratory failure (as patients with specific malignancies could have increased CEA levels without any correlation with COVID-19, all patients with primary malignancy were excluded from the study); (5) patients whose survival time, endpoint (overall survival), demographic information or treatment data was unknown; and (6) patients whose admission carcinoembryonic antigen (CEA) was unknown.

The clinical data in the study were retrieved from the electronic medical record system of Wuhan Jinyintan Hospital on initial admission, including variables of demographic information (age at diagnosis and gender), symptom (fever, cough, expectoration, shortness of breath and diarrhea), comorbidity (diabetes mellitus, hypertension, cardiovascular disease and cerebral infarction hypertension) and therapeutic information (use of glucocorticoid, imaging score, nasal catheter, high flow oxygen intake, ventilation). Additionally, laboratory indexes were also collected including CEA (ng/ml), albumin (g/l), hemoglobin (g/l), neutrophils (× 10^9^/l), lymphocytes (× 10^9^/l), C-reactive protein (CRP, mg/l), hypokalemia, hypocalcemia, hyponatremia, hyperkalemia and hypernatremia. The cutoff values of laboratory and imaging indexes were determined according to the normal values stipulated by the laboratory and imaging department of Wuhan Jinyintan Hospital.

As the endpoint, the survival time and overall survival status of each patient were retrieved. The endpoint of the present study was the overall death of COVID-19 patient, which presented the outcome and prognosis of patients in this study. Patients who were diagnosed after April 30, 2020, were excluded from the study.

### Epidemiological statistical analysis

The retrospective study started with descriptive statistic: Discontinuous variables were presented as percentages while continuous variables in normal distribution were described as mean ± standard deviation (SD) or else reported as median (range). Two statistical methods were applied to explore potential significant predictors. As initial parameter or nonparametric tests, the Chi-square test was used to compare the outcomes between discontinuous variables, and variance homogeneous and normal distributed continuous variables were compared by the Student t-test; otherwise, the Mann–Whitney U-test or Kruskal–Wallis H-test was used. Besides, the Kaplan–Meier survival analysis was used to determine the prognostic value of each variable. Furthermore, predictors with statistical significance in both parameter or nonparametric tests and Kaplan–Meier survival analysis were selected to construct the multivariate Cox proportional hazard model. The nomogram was established based on the multivariate model to predict the prognosis of COVID-19 patients. The significant prognostic factors in multivariate Cox model were marked with asterisks (*) in the nomogram (*: P < 0.05; **: P < 0.01). Receiver operating characteristic (ROC) curve and calibration curve were drawn to evaluate the discrimination and calibration of the nomogram.

### Processing of single-cell RNA-seq data

Single-cell RNA-sequence (scRNA-seq) data of COVID-19 patients’ and healthy volunteer’s bronchoalveolar lavage fluid (BALF, accession no. GSE145926) [15] and peripheral blood mononuclear cells (PBMCs, accession no. GSE150728) [16] were download from the Gene Expression Omnibus (GEO). All BALF and PBMC samples were taken at the initial admission.

The preliminary data processing of single-cell RNA-seq data started from the Cell Ranger Single Cell Software Suite 3.3.1 (http://10xgenomics.com/). The pair-ended reads fastq files were trimmed to remove template switch oligo (TSO) sequence and poly-A tail sequence. Then, command of “cellranger count” was used to quantify the clean reads, aligned to the hg38 human genome. The Seurat method was applied to integrated data analysis [17].

In terms of quality control (QC), genes with average read count greater than one and being expressed in at least three single cells were considered for further analysis. Cells with either fewer than 100,000 transcripts or fewer than 1,500 genes were filtered out.

In data processing, first, variance stabilizing transformation (VST) method was used to identify variable genes. Variable genes were input as initial features for principal component analysis (PCA) [17]. Then, the principal components (PCs) with P values < 0.05 were filtered by the jackstraw analysis and were incorporated into further UMAP (uniform manifold approximation and projection) and t-distributed stochastic neighbor embedding (t-SNE) to identify cell subclusters (resolution = 0.50) [18]. Only the genes with |log2 fold change (FC)|> 0.5 and false discovery rate (FDR) value < 0.05 were identified as differentially expressed genes (DEGs) among cell subclusters. Feature plots and violin plots were utilized to illustrate the distribution and expression of DEGs, respectively. Additionally, scMatch [19], singleR [20] and CellMarker [21] were used as references to define each cluster. Cell trajectory and pseudo-time analysis was performed by monocle2 [22]. Furthermore, 50 hallmark gene sets were retrieved from the Molecular Signatures Database (MSigDB) version 7.1 (https://www.gsea-msigdb.org/gsea/msigdb/index.jsp) and gene set variation analysis (GSVA) algorithm was performed to absolutely quantify the activity of signaling pathways in each single cell [23, 24]. Furthermore, the CellphoneDB algorithm was used to identify the cellular communication between pneumonocyte and immune cells [25].

### Identification of the mechanism of abnormal CEA expression in COVID-19 patients

First of all, the distribution and expression of CEA-related genes (CRGs) including CEACAM1, CEACAM3, CEACAM4, CEACAM5, CEACAM6, CEACAM7, CEACAM8, CEACAM16, CEACAM18, CEACAM19, CEACAM20, CEACAM21, CEACAMP1, CEACAMP2, CEACAMP3, CEACAMP4, CEACAMP5, CEACAMP6, CEACAMP7, CEACAMP8, CEACAMP9, CEACAMP10, CEACAMP11 and CEACAM22P were visualized by feature plot and violin plot in BALF and PBMC scRNA-seq data. Then, co-expression (correlation) analysis was performed among CRGs and 50 hallmark of gene sets to identify the potential downstream pathways. The CellphoneDB algorithm was used to illuminate the cellular communication between cells with high CRG expression and other cells. Besides, two data including scRNA-seq data of acute lung injury (ALI) mouse lung (GSE134383) and idiopathic pulmonary fibrosis (IPF) mouse lung (E-HCAD-14) were downloaded to evaluate the distribution and expression of CRGs, key receptor–ligand pair of cellular communication and potential downstream pathways [26–30].

### Statistical analysis

Only p value of two-sided statistical testing lower than 0.05 was considered statistically significant. All statistical analysis processes were performed with R version 3.6.1 software (Institute for Statistics and Mathematics, Vienna, Austria; www.r-project.org).

## Results

### Patient characteristics and univariate analysis

A total of 300 hospitalized adult COVID-19 patients diagnosed by RT-PCR in Wuhan Jinyintan Hospital from January 1, 2020, to April 30, 2020, were included in this retrospective study.

**Fig. 1:**
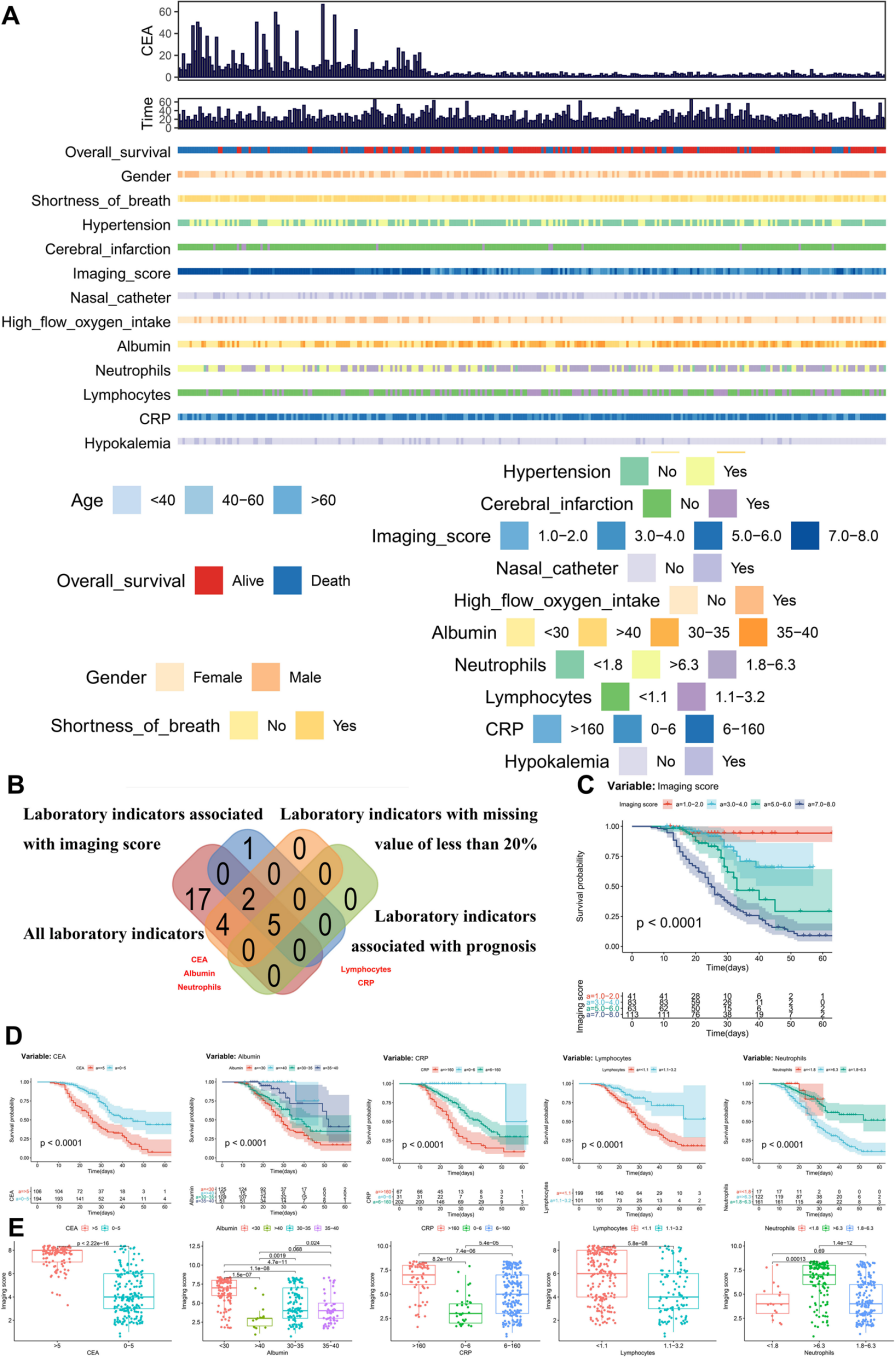
Patient characteristics and univariate analysis. The baseline characteristics of 300 COVID-19 patients were described in (**A**). The cohort comprised 170 males and 130 females, with a median age of 63.0 (range 21.0–90.0) years. After removing 17 of the 28 laboratory indicators with missing values more than 20% of the sample size, the results of initial Kaplan–Meier survival analysis (**C**–**D**) and parameter or nonparametric tests (**E**) suggested that only five (serum CEA, lymphocytes, neutrophils, CRP and albumin) indicators were significantly associated with both imaging score and prognosis COVID-19 patients (**B**)

The baseline characteristics of 300 COVID-19 patients are described in Fig. 1A and Table 1. The cohort comprised 170 males and 130 females, with a median age of 63.0 (range 21.0–90.0) years. After removing 17 of 28 laboratory indicators with missing values more than 20% of the sample size, the results of initial Kaplan–Meier survival analysis (Fig. 1C–D) and parameter or nonparametric tests (Fig. 1E) revealed that only five indicators (serum CEA, lymphocytes, neutrophils, CRP and albumin) were significantly associated with both the imaging score and prognosis of COVID-19 patients (Fig. 1B).

**Table 1:** Baseline characteristics of COVID-19 patients

	Total patients (N = 300)	
	No	%
*Age (year)*		
Median (range)	45.0(0 ~ 93)	
Average ± SD		
*Categorical age*		
< 40	26	8.7
40–60	110	36.7
> 60	164	54.7
*Gender*		
Female	130	43.3
Male	170	56.7
*Fever*		
No	25	8.3
Yes	275	91.7
*Cough*		
No	75	25
Yes	225	75
*Expectoration*		
No	221	73.7
Yes	79	26.3
*Shortness of breath*		
No	128	42.7
Yes	172	57.3
*Diarrhea*		
No	293	97.7
Yes	7	2.3
*Comorbidities*		
No	256	85.3
Yes	44	14.7
*Diabetes mellitus*		
No	251	83.7
Yes	49	16.3
*Hypertension*		
No	193	64.3
Yes	107	35.7
*Cardiovascular disease*		
No	272	90.7
Yes	28	9.3
*Cerebral infarction*		
No	286	95.3
Yes	14	4.7
*Glucocorticoid*		
No	272	90.7
Yes	28	9.3
*Imaging score*		
1.0–2.0	41	13.7
3.0–4.0	83	27.7
5.0–6.0	63	21
7.0–8.0	113	37.7
*Nasal catheter*		
No	141	47
Yes	159	53
*High flow oxygen intake*		
No	216	72
Yes	84	28
*Noninvasive ventilation*		
No	238	79.3
Yes	62	20.7
*Invasive ventilation*		
No	222	74
Yes	78	26
*Mode of ventilation*		
No oxygen	47	15.7
OWNC	99	33.0
HFNC	33	11.0
NIV	38	12.7
IV	83	27.6
*Stage*		
Mild	47	15.7
Severe	132	44.0
Critical	121	40.3
*CEA (ng/ml)*		
Median (range)	3.25 (0.50 ~ 66.60)	
Average ± SD	6.88 ± 10.20	
*Categorical CEA*		
0–5	194	64.7
> 5	106	35.3
*Albumin (g/l)*		
< 30	125	41.7
30–35	109	36.3
35–40	51	17
> 40	15	5
*Hemoglobin (g/l)*		
< 115	108	36
115–150	182	60.7
> 150	10	3.3
*Neutrophils (*× *10*^*9*^*/l)*		
< 1.8	17	5.7
1.8–6.3	161	53.6
> 6.3	122	40.7
*Lymphocytes (*× *10*^*9*^*/l)*		
< 1.1	199	66.3
1.1–3.2	101	33.7
*CRP (mg/l)*		
0–6	31	10.3
6–160	202	67.3
> 160	67	22.3
*Hypokalemia*		
No		85.7
Yes	43	14.3
*Hypocalcemia*		
No	138	46
Yes	162	54
*Hyponatremia*		
No	275	91.7
Yes	25	8.3
*Hyperkalemia*		
No	287	95.7
Yes	13	4.3
*Hypernatremia*		
No	295	98.3
Yes	5	1.7
*Overall survival*		
Alive	174	58.0
Dead	126	42.0
*Survival time (day)*		
Median (Range)	24 (6 ~ 66)	
Average ± SD	26.60 ± 11.09	

### Cox proportional hazard model and nomogram

**Fig. 2:**
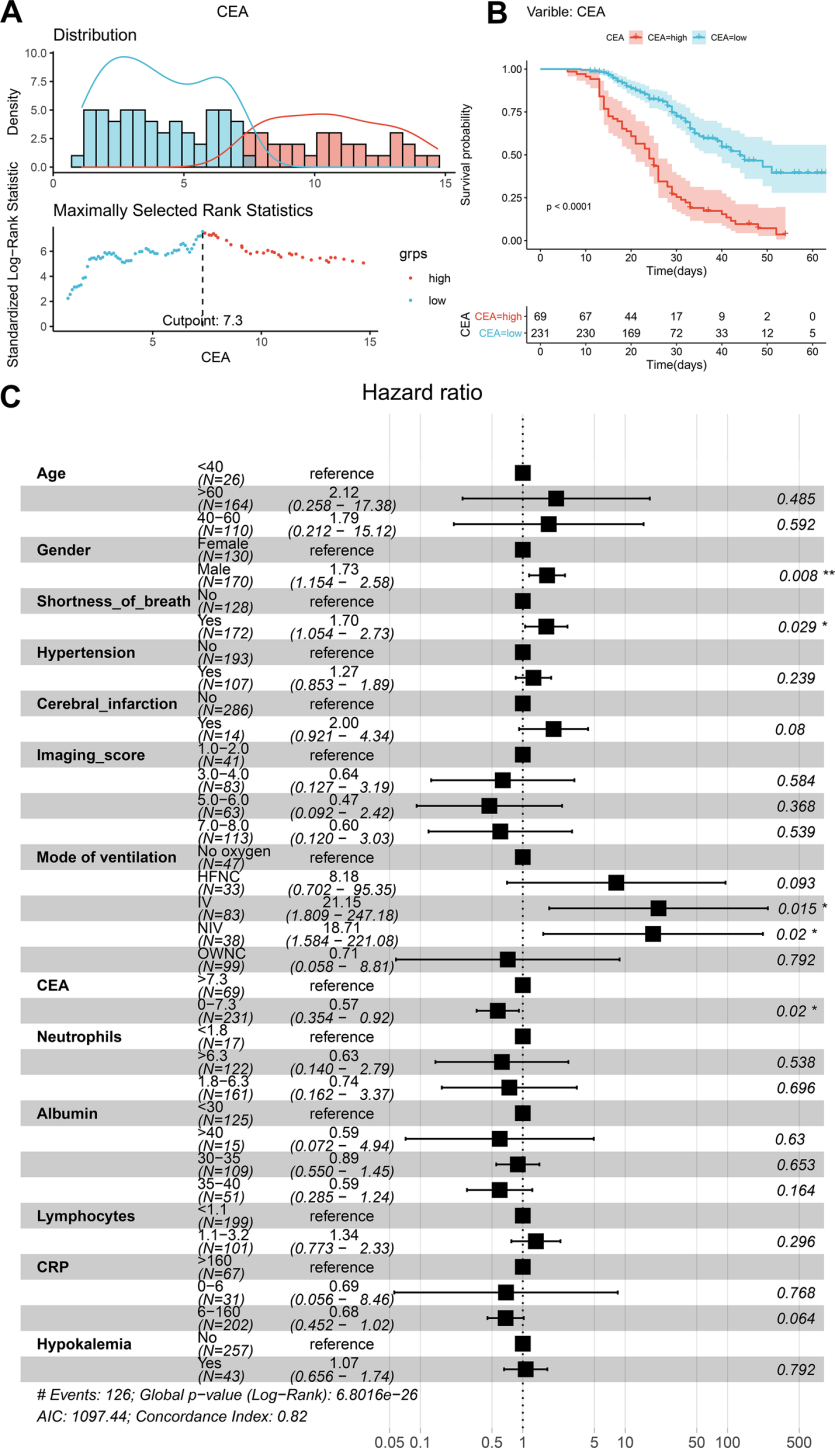
Cox proportional hazard model. CEA is the only laboratory indicator with significant results in all univariate and multivariate analyses. To identify the optimal cutoff point of CEA, the cyclic log-rank test was performed. And the results showed that CEA = 7.3 ng/ml was the optimal cutoff point with the most significant P value in log-rank test (**A**, **B**). Then, 12 potential significant indicators (showing prognostic values in Kaplan–Meier analysis) and two demographic information (age and gender) were incorporated into the initial Cox proportional hazard models, and the final multivariate models were constructed to confirm the effects of significant covariates in the initial models to the OS of COVID-19 patients (**C**). The variable of nasal catheter is integrated into a new variable named "Mode of ventilation," a variable with five levels (no oxygen; oxygen with nasal canula (OWNC); oxygen through high flow nasal canula (HFNC); noninvasive ventilation (NIV); and invasive ventilation (IV)). The results suggested that patients with lower CEA had better OS (HR 0.57; 95% CI 0.354 to 0.920; P = 0.020) in multivariate model, which suggested that CEA independently prognostic indicator for COVID-19 patients

CEA is the only laboratory indicator with significant difference in all the univariate and multivariate analysis. To identify the optimal cutoff point of CEA, the cyclic log-rank test was conducted. The results revealed that CEA = 7.3 ng/ml was the optimal cutoff point with the most significant P value in the log-rank test (Fig. 2A, B). The variable of nasal catheter is integrated into a variable named “Mode of ventilation,” which is a variable with five levels (no oxygen; oxygen with nasal canula (OWNC); oxygen through high flow nasal canula (HFNC); noninvasive ventilation (NIV); and invasive ventilation (IV)). Since mode of ventilation has the property of the ordered categorical variable, even if a patient only takes HFNC on admission, then the condition deteriorates and he or she receives invasive ventilation, the variable "Mode of ventilation" will be marked as IV but not HFNC. Then, 11 potential indicators showing prognostic values in Kaplan–Meier analysis were incorporated into the initial Cox proportional hazard models, along with two demographic information (age and gender). The final multivariate models were constructed to confirm the effects of significant covariates in the initial models of the overall survival (OS) of COVID-19 patients (Fig. 2C and Table 2). Patients with lower CEA had better OS (HR 0.57; 95% CI 0.354 to 0.920; P = 0.020) in the multivariate model, suggesting CEA as a prognostic indicator for COVID-19 patients independently.

**Table 2:** Cox proportional hazard regression model for overall survival of COVID-19 patients

Variable	Overall survival (OS)	
	Hazard ratio (95% CI)	P
*Categorical age*		
< 40	1.00 (reference)	
40–60	1.791 (0.212 to 15.119)	0.592
> 60	2.118 (0.258 to 17.384)	0.485
*Gender*		
Female	1.00 (reference)	
Male	1.727 (1.154 to 2.585)	0.008*
*Shortness of breath*		
No	1.00 (reference)	
Yes	1.697 (1.054 to 2.731)	0.029*
*Hypertension*		
No	1.00 (reference)	
Yes	1.271 (0.853 to 1.893)	0.239
*Cerebral infarction*		
No	1.00 (reference)	
Yes	1.999 (0.921 to 4.342)	0.080
*Imaging score*		
1.0–2.0	1.00 (reference)	
3.0–4.0	0.638 (0.127 to 3.191)	0.584
5.0–6.0	0.472 (0.092 to 2.421)	0.368
7.0–8.0	0.603 (0.120 to 3.027)	0.539
*Mode of ventilation*		
No oxygen	1.00 (reference)	
OWNC	0.713 (0.058 to 8.810)	0.792
HFNC	8.181 (0.702 to 95.353)	0.093
NIV	18.714 (1.584 to 221.082)	0.020*
IV	21.148 (1.809 to 247.183)	0.015*
*Categorical CEA*		
> 7.3	1.00 (reference)	
0–7.3	0.570 (0.354 to 0.917)	0.020*
*Albumin (g/l)*		
< 30	1.00 (reference)	
30–35	0.894 (0.550 to 1.454)	0.653
35–40	0.594 (0.285 to 1.240)	0.164
> 40	0.595 (0.072 to 4.937)	0.630
*Neutrophils (*× *10*^*9*^*/l)*		
< 1.8	1.00 (reference)	
1.8–6.3	0.739 (0.162 to 3.370)	0.696
> 6.3	0.630 (0.140 to 2.788)	0.538
*Lymphocytes (*× *10*^*9*^*/l)*		
< 1.1	1.00 (reference)	
1.1–3.2	1.343 (0.773 to 2.332)	0.296
*CRP (mg/l)*		
> 160	1.00 (reference)	
0–6	0.686 (0.056 to 8.457)	0.768
6–160	0.683 (0.452 to 1.023)	0.064
*Hypokalemia*		
No	1.00 (reference)	
Yes	1.068 (0.656 to 1.737)	0.792

**Fig. 3:**
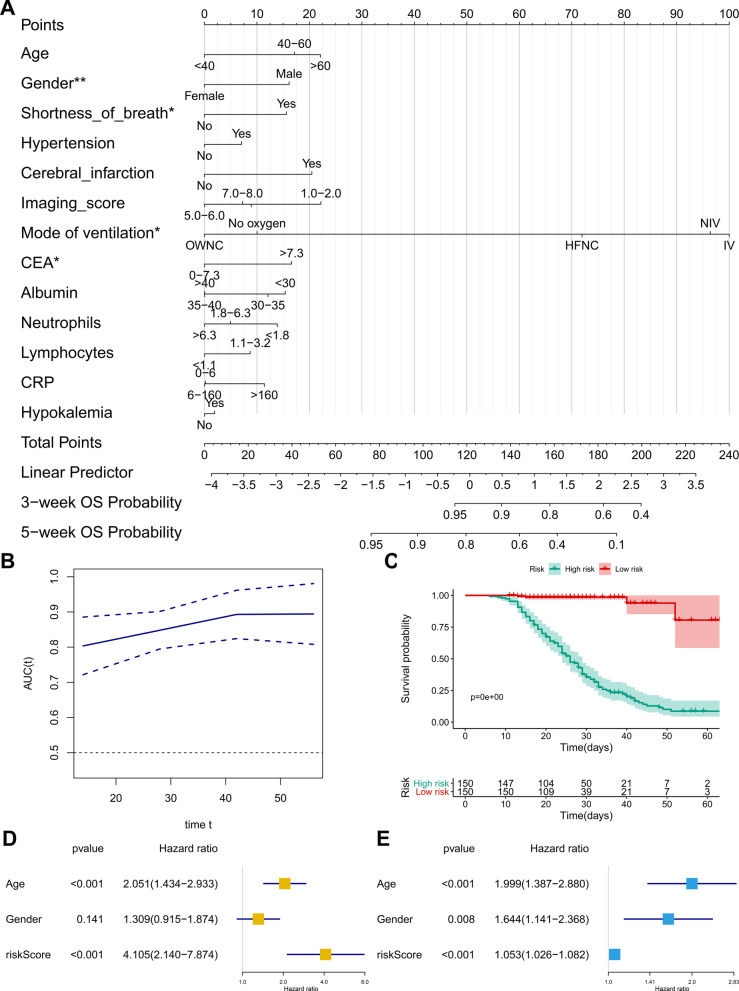
Construction and model diagnosis of prognostic nomogram. The prognostic nomogram was constructed based on the multivariate Cox model including CEA, which could predict the 3-week and 5-week overall survival probability of COVID-19 patients (**A**). The significant prognostic factors in multivariate Cox model were marked with asterisks (*) in the nomogram (*: P < 0.05; **: P < 0.01). The time-related ROC curve suggested acceptable discrimination of the nomogram (**B**). Besides, the risk score (RS) was calculated by the formula generated by the multivariate Cox model. Kaplan–Meier curve suggested the prognostic value of the RS (**C**, P < 0.001). Eventually, in univariate (HR = 4.105, 95% CI (2.140 − 7.874), P < 0.001) (**D**) and multivariate (HR = 1.053, 95% CI (1.026 − 1.082), P < 0.001) (**E**) Cox regression model corrected by demographics, the RS was shown to be an independent prognostic indicator for COVID-19 patients

The prognostic nomogram was constructed based on the multivariate Cox model including CEA to predict the 3-week and 5-week overall survival probability of COVID-19 patients (Fig. 3A). The calibration curve and the ROC curve (AUC = 0.783) suggested acceptable calibration and discrimination of the nomogram, respectively (Fig. 3B; Additional file 5: Figure S1A-B). Besides, the risk score (RS) was calculated by the formula generated by the multivariate Cox model. The scatter plot (Additional file 5: Figure S1C) and risk curve (Additional file 5: Figure S1D) of the model demonstrated the RS distribution based on risk score of each patient. The Kaplan–Meier curve suggested the prognostic value of the RS (Fig. 3C, P < 0.001). Additionally, the residual distribution of the multivariate model was accessed by the residual plot (Additional file 5: Figure S1E). Eventually, the RS was shown to be an independent prognostic indicator for COVID-19 patients in both univariate (HR = 4.105, 95% CI (2.140 − 7.874), P < 0.001, Fig. 3D) and multivariate (HR = 1.053, 95% CI (1.026 − 1.082), P < 0.001, Fig. 3E) Cox regression model corrected by demographics.

Additionally, since many laboratory values were not analyzed as there were more than 20% missing data for 17 out of 28 variables, raising some concern on the fact some prognosis factors could remain unidentified, the results of Kaplan–Meier analysis of 17 laboratory values with more than 20% missing data were illustrated by survival curves (Additiona file 1: Table S1; Additional file 5: Figure S1F). Some laboratory indicators, such as ALT (alanine aminotransferase), AST (aspartate aminotransferase), PLT (platelet), PCT (procalcitonin), HBDH (α-hydroxybutyrate dehydrogenase), LDH (lactate dehydrogenase), ferritin, IL-6 (interleukin 6), D-dimer, myoglobin and HsTNT (high-sensitivity troponin) did show prognostic values in univariate analyses.

In order to further prove whether these laboratory indicators with significance in univariate analysis can be used as independent prognostic factors, we, respectively, incorporated these variables into the multivariate Cox regression model and conducted multivariate Cox regression analysis for 22 times (keep or remove missing values). In the cohort where missing values were removed, only the regression models, respectively, including AST, ALT, D-dimer and PLT were converged (due to the uneven distribution of some variables level and events number when removing missing values), suggesting that CEA was an independent prognostic factor in all multivariate models and both normal PLT (HR = 0.635, 95% CI (0.408 to 0.990), P = 0.045) and normal ferritin (HR = 0.094, 95% CI (0.010 to 0.860), P = 0.037) were also independent favorable factors (Additiona file 2: Supplementary material 1) compared abnormal levels. In the cohort keeping missing values, a total of 11 regression models were converged and CEA was an independent prognostic factor in all multivariate models. Additionally, patients with normal PLT (HR 0.624; 95% CI 0.406 to 0.960; P = 0.031), ferritin (HR 0.089; 95% CI 0.010 to 0.750; P = 0.026), IL − 6 (HR 0.494; 95% CI 0.264 to 0.930; P = 0.028) and myoglobin (HR 0.520; 95% CI 0.303 to 0.890; P = 0.017) had better OS than patients with abnormal levels of these laboratory indicators in the multivariate models (Additiona file 3: Supplementary material 2). However, most of these laboratory indicators did not meet the requirements for inclusion in multivariate analysis [31, 32]. Inclusion of more covariates does not necessarily lead to higher accuracy, but instead to overfitting, and should be avoided. Thus, only five indicators (serum CEA, lymphocytes, neutrophils, CRP and albumin) were incorporated into the multivariate Cox model. And all these results suggested CEA as a prognostic indicator for COVID-19 patients independently.

### Subgroup analysis

As smokers or patients with specific malignancies could have increased CEA levels without any correlation with COVID-19, all patients with primary malignancy were excluded from the study. Furthermore, in order to identify the association between CEA levels and smoking, two subgroup Cox proportional hazard regression models including smoking status (keep or remove missing values) were constructed, suggesting that the CEA level (HR 0.547; 95% CI 0.318 to 0.940; P = 0.037) (remove missing values) (HR 0.620; 95% CI 0.384 to 0.990; P = 0.048) (keep missing values) was still an independent prognostic indicator for COVID-19 patients (SAdditiona file 4: Supplementary material 3). Moreover, CRG expression levels were retrieved from the RNA-seq data of lung squamous cell carcinoma (LUSC) available from The Cancer Genome Atlas (TCGA). The results of rank-sum tests showed that CEA levels in both the serum of COVID-19 patients (Additional file 6: Figure S2A) and the tissues of lung cancer (Additional file 6: Figure S2B-C) were significantly higher in smokers than in non-smokers.

Additionally, to further evaluate the prognostic value of the mode of ventilation, the Kaplan–Meier analysis was performed. The results suggested that the mode of ventilation was significantly associated with the prognosis of COVID-19 patients (P < 0.001) (Additional file 7: Figure S3).

### Identification of the potential mechanism of CEA in COVID-19

**Fig. 4:**
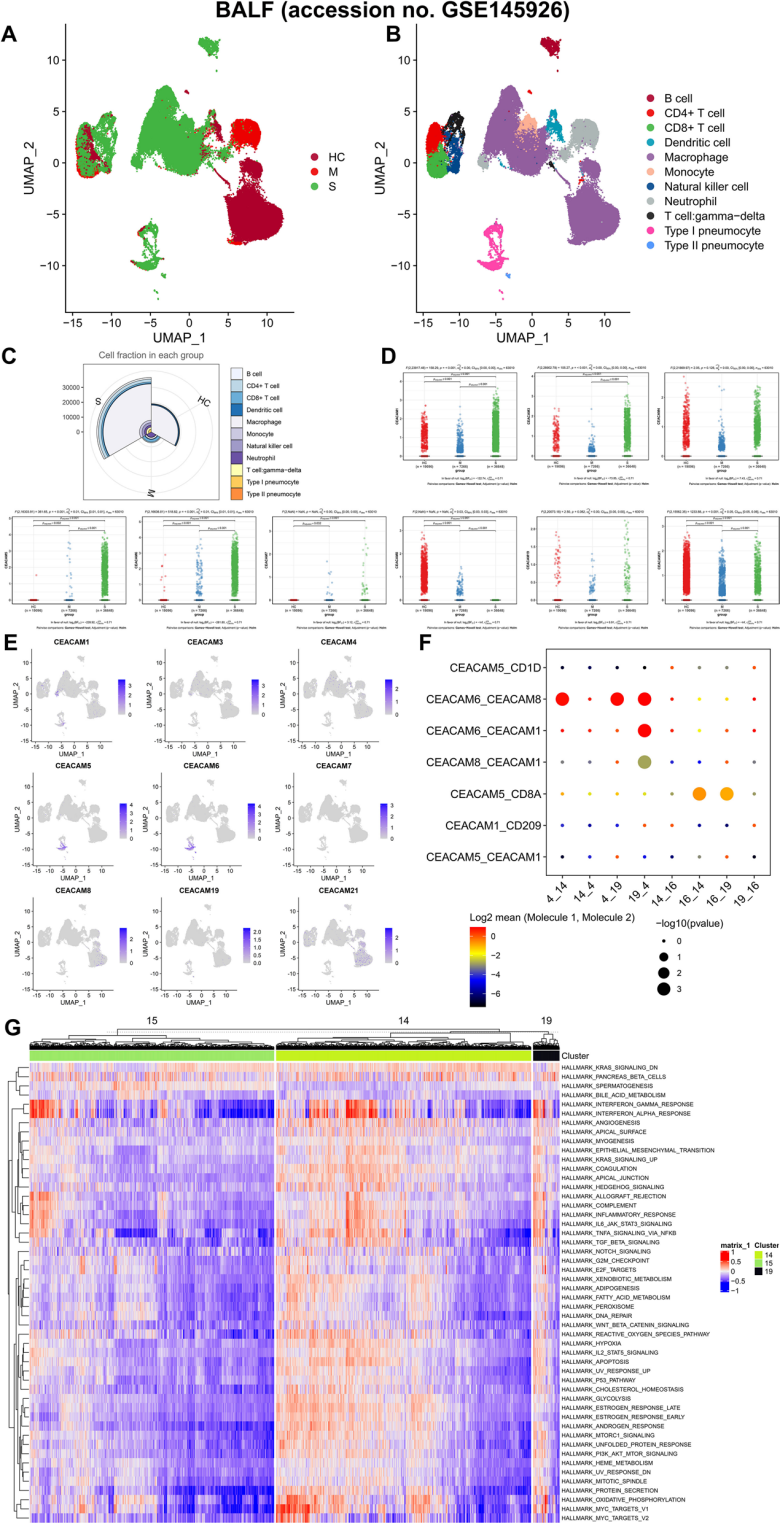
Identification of the mechanism of abnormal CEA expression in COVID-19 patients’ and healthy volunteers’ bronchoalveolar lavage fluid (BALF). scRNA-seq data of bronchoalveolar lavage fluid (BALF) from three patients with moderate COVID-19 (C141, C142 and C144), six patients with severe or critical infection (C143, C145, C146, C148, C149 and C152) and three healthy controls (C51, C52 and C100) (accession no. GSE145926) were download from the GEO database. A UAMP analysis was performed in 63,010 cells in BALF and clearly identified 20 clusters and 11 cell types (B cell, CD4 + T cell, CD8 + T cell, dendritic cell, macrophage, monocyte, natural killer cell, neutrophil, T cell: gamma–delta, type I pneumocyte, type II pneumocyte) (**A**, **B**). All other immune cells (B cell, CD4 + T cell, CD8 + T cell, dendritic cell, monocyte, natural killer cell, neutrophil and T cell: gamma–delta) except for macrophages and type I and type II pneumocytes were dominantly differentiated and chemotactic in COVID-19 patients’ BALF compared to healthy volunteer’s BALF (**C**). Furthermore, in terms of the expression and distribution of CRGs, CEACAM1, CEACAM3, CEACAM5, CEACAM6, CEACAM7, CEACAM8 and CEACAM21 were differentially expressing among moderate, severe/critical COVID-19 patients and healthy controls while CEACAM5 and CEACAM6 were significantly localized in the type II pneumocytes of COVID-19 patients (**D**, **E**). In particular, **F** summarized the absolute quantification of 50 hallmark gene sets calculated the GSVA in type I and type II pneumocytes, suggesting that the interferon response and cell proliferation signaling pathways were significantly activated in type II pneumocytes highly expressing CRGs of COVID-19 patients (**F**)

The scRNA-seq data of bronchoalveolar lavage fluid (BALF) from three patients with moderate COVID-19 (C141, C142 and C144), six patients with severe or critical infection (C143, C145, C146, C148, C149 and C152) and three healthy controls (C51, C52 and C100) [15] were download from the GEO database. (This part was a secondary analysis of published data.) A UAMP analysis was performed in 63,010 cells in BALF and clearly identified 20 clusters and 11 cell types including B cell, CD4 + T cell, CD8 + T cell, dendritic cell, macrophage, monocyte, natural killer cell, neutrophil, T cell: gamma–delta, type I pneumocyte, type II pneumocyte (Fig. 4A, B, Additional file 8: Figure S4A-B). The expression levels and expression percentages of the marker genes in each cell type were displayed in Additional file 8: Figure S4C and S4D, respectively. Except for macrophages and type I and type II pneumocytes, all other immune cells (B cell, CD4 + T cell, CD8 + T cell, dendritic cell, monocyte, natural killer cell, neutrophil and T cell: gamma–delta) were dominantly differentiated and chemotactic in the BALF of COVID-19 patients compared to healthy volunteer (Fig. 4C). Furthermore, in terms of the expression and distribution of CRGs, CEACAM1, CEACAM3, CEACAM5, CEACAM6, CEACAM7, CEACAM8 and CEACAM21 were differentially expressed among moderate, severe/critical COVID-19 patients and healthy controls while CEACAM5 and CEACAM6 were significantly localized in the type II pneumocytes of COVID-19 patients (Fig. 4D–E). Additionally, the cell cycle analysis suggested that COVID-19 patients were more likely to have cells in the G2M and S stages (Additional file 9: Figure S5A-B). Moreover, cellphoneDB analysis illustrated that pneumocytes of COVID-19 patients communicated extensively with other immune cells through CRGs. In particular, the number of type II pneumocyte was found to significantly increase in COVID-19 and have cross talk with neutrophils via CEACAM8-CEACAM6 (Fig. 4F, Additional file 9: Figure S5C). Figure 4G summarizes the absolute quantification of 50 hallmark gene sets calculated the GSVA in type I and type II pneumocytes, suggesting that the interferon response and cell proliferation signaling pathways were significantly activated in type II pneumocytes highly expressing CRGs of COVID-19 patients.

**Fig. 5:**
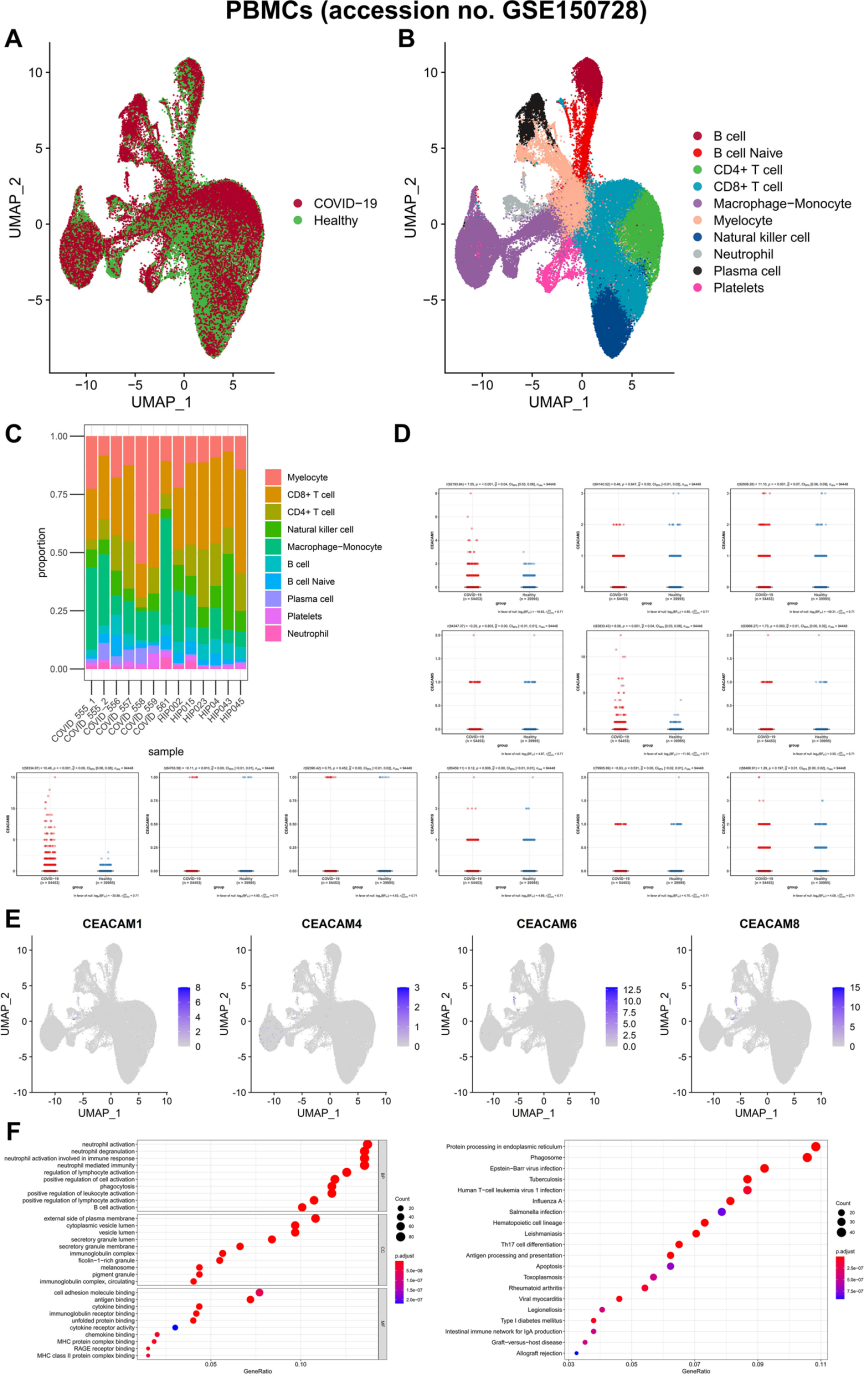
Identification of the mechanism of abnormal CEA expression in COVID-19 patients’ and healthy volunteers’ PBMCs scRNA-seq data of 94,448 PBMCs from six patients with moderate COVID-19 and six healthy volunteers were download from the GEO database (accession no. GSE150728). The UAMP analysis identified 18 clusters and 10 cell types (B cell, B cell Naïve, CD4 + T cell, CD8 + T cell, macrophage–monocyte, myelocyte, natural killer cell, neutrophil, plasma cell, olatelets) (**A**, **B**). All types of immune cell were significantly differentiated and chemotactic in COVID-19 patients’ PBMCs compared to healthy controls (**C**). What is more, CEACAM1, CEACAM4, CEACAM6 and CEACAM8 were differentially expressing between PBMCs of COVID-19 patients and healthy controls while CEACAM1, CEACAM6 and CEACAM8 were significantly localized in a novel cell subtype annotated as “developing neutrophils,” which was significantly differentiated and chemotactic only in COVID-19 patients with ARDS reported by Wilk, A.J., et al. (**D**, **E**). Additionally, dot plots in **F** summarized the results of Gene Ontology (GO) and the Kyoto Encyclopedia of Genes and Genomes (KEGG) enrichment analysis of the DEGs of the developing neutrophils (**F**)

Similarly, the scRNA-seq data of 94,448 PBMCs from six patients with moderate COVID-19 and six healthy volunteers were also downloaded (This part was a secondary analysis of published data) [16]. The UAMP analysis identified 18 clusters and 10 cell types including B cell, B cell Naïve, CD4 + T cell, CD8 + T cell, macrophage–monocyte, myelocyte, natural killer cell, neutrophil, plasma cell. platelets (Fig. 5A, B; Additional file 10: Figure S6A-B). All types of immune cell were significantly differentiated and chemotactic in COVID-19 patients’ PBMCs compared to healthy controls (Fig. 5C). CEACAM1, CEACAM4, CEACAM6 and CEACAM8 were differentially expressed between PBMCs of COVID-19 patients and healthy controls, while CEACAM1, CEACAM6 and CEACAM8 were significantly localized in a novel cell subtype annotated as developing neutrophils, which was significantly differentiated and chemotactic (Fig. 5D, E). Additionally, dot plots summarized the results of Gene Ontology (GO) and the Kyoto Encyclopedia of Genes and Genomes (KEGG) enrichment analysis. Based on GO analysis, the DEGs were associated with the neutrophil activation and degranulation (Fig. 5F). According to KEGG analysis, the DEGs were related to protein processing in endoplasmic reticulum, phagosome, Epstein–Barr virus infection and tuberculosis (Fig. 5F). Additionally, cell cycle analysis suggested that the developing neutrophils in COVID-19 patients’ PBMCs were all engaged in the G2M and S stages (Additional file 10: Figure S6C-D). And more extensive cellular communication analysis performed by iTALK algorithm (https://github.com/Coolgenome/iTALK/) further illustrated mechanisms between the developing neutrophils and the other PBMCs (Additional file 10: Figure S6E-F). Eventually, all neutrophils were extracted separately and re-analyzed for dimensionality reduction. The UAMP analysis identified two cell types including canonical neutrophils and developing neutrophils (Additional file 11: Figure S7A-B). A significant increase in the number of developing neutrophils was found in COVID-19 while CEACAM1, CEACAM6 and CEACAM8 were also significantly co-localized developing neutrophils (Additional file 11: Figure S7C-D).

### The specific expressions of CRGs in COVID-19 patients

**Fig. 6:**
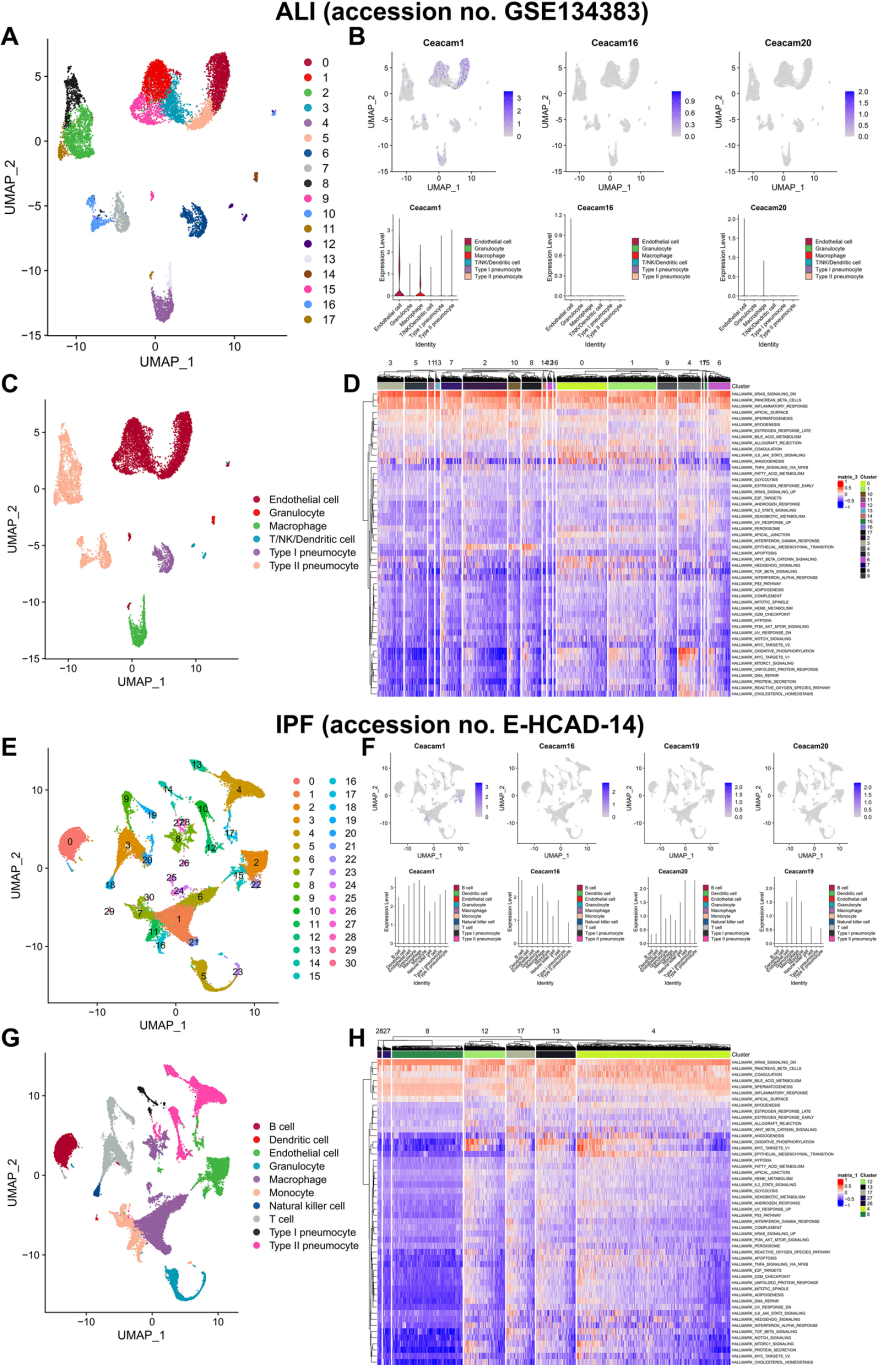
The abnormal expressions of CRGs in COVID-19 patients were COVID-19-specific and not related to CEA involvement in ALI and IPF. Due to the close correlation between CEA and ALI and IPF, we initially speculated that the poor prognosis of COVID-19 patients mediated by CEA might be related to ALI and IPF pathophysiologically. To validate this hypothesis, scRNA-seq data of ALI and IPF mouse lungs were also downloaded to evaluated the distribution and expression of CRGs, key receptor–ligand pair of cellular communication and potential downstream pathways. The UAMP analysis identified 18 clusters and 6 cell types in ALI mouse lungs while there were no abnormal expressions of CRGs (**A**, **C**). And the interferon response and cell proliferation signaling pathways were not significantly activated in type II pneumocytes of ALI mouse lungs (**D**). Similarly, abnormal expressions of CRGs were also not detected in 31 clusters and 10 cell types of IPF mouse lungs (**E**, **G**). Besides, the heatmap of GSVA also showed that the interferon response and cell proliferation signaling pathways were not activated in type II pneumocytes of IPF mouse lungs (**H**)

The scRNA-seq data of ALI mouse lungs (https://www.ncbi.nlm.nih.gov/geo/query/acc.cgi?acc=GSE134383) and IPF mouse lungs (https://www.ebi.ac.uk/gxa/sc/experiments/E-HCAD-14/results/tsne) were also downloaded to evaluate the distribution and expression of CRGs, key receptor–ligand pair of cellular communication and potential downstream pathways [26–30]. The UAMP analysis identified 18 clusters and 6 cell types in ALI mouse lungs while there were no abnormal expressions of CRGs (Fig. 6A–C). The interferon response and cell proliferation signaling pathways were not significantly activated in type II pneumocytes of ALI mouse lungs (Fig. 6D). Similarly, abnormal expressions of CRGs were also not detected in 31 clusters and 10 cell types of IPF mouse lungs (Fig. 6E–G). Besides, the heatmap of GSVA also showed that the interferon response and cell proliferation signaling pathways were not activated in type II pneumocytes of IPF mouse lungs (Fig. 6H). Therefore, abnormal expressions of CRGs in COVID-19 patients were COVID-19-specific and not related to CEA involvement in ALI and IPF.

### Protein–protein interaction (PPI) network of CRGs

**Fig. 7:**
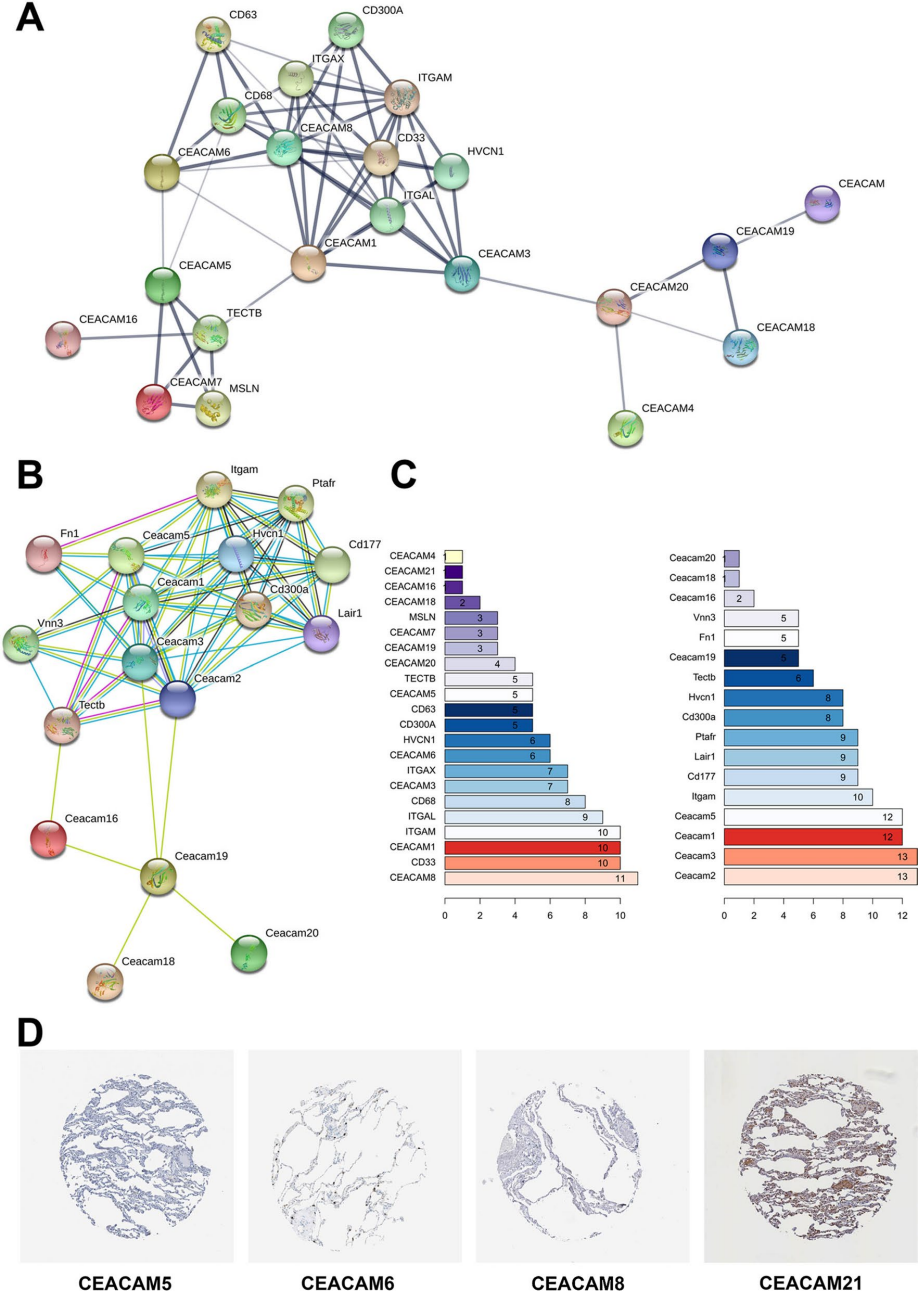
Protein–protein interaction (PPI) network of CRGs. String database was used to construct the PPI network of CRGs, illustrating that several CRGs had direct protein–protein interactions with a variety of immune cell surface markers (**A**, **C**). Besides, the protein expression levels of CRGs in normal lung samples of The Human Protein Atlas were also checked, showing that only CEACAM21 were stained moderately in pneumocytes while the proteins of CEACAM5, CEACAM6 and CEACAM8 were not detected in normal lung samples (**D**)

String [33] database was utilized to construct the PPI network of CRGs, illustrating that several CRGs had direct interaction with a variety of immune cell surface markers (Fig. 7A–C). Besides, the protein expression levels of CRGs in normal lung samples of The Human Protein Atlas were also checked [34], showing that only CEACAM21 was stained moderately in pneumocytes while the proteins of CEACAM5, CEACAM6 and CEACAM8 were not detected in normal lung samples (Fig. 7D). To sum up, the prognostic value of CEA was identified in COVID-19 patients and the developing neutrophils/neutrophil progenitors (highly expressed CEACAM8, ELANE and LYZ) could have the cross talk with type II pneumocyte (highly expressed CEACAM5 and CEACAM6) via CEACAM8-CEACAM6.

## Discussion

The COVID-19 has induced a worldwide epidemiological event with a high infectivity and mortality [35]. Identification of predicting biomarkers may assist clinicians in decision-making. However, the candidate predictors of COVID-19 remain unclear. In this study, we identified CEA as a potential biomarker for COVID-19 patients. To further explore the underlying mechanism, we used the single-cell transcriptomics of BALF from patients with or without COVID-19, along with the scRNA-seq data of ALI and IPF mouse lungs. We found that the developing neutrophils/neutrophil progenitors can have the cross talk with type II pneumocyte via CEACAM8-CEACAM6 in COVID-19 but not ALI and IPF.

The predicting biomarkers are important for clinical decision-making; thus, many efforts have been made to identify them in patients with COVID-19 pneumonia. Previously, the inflammatory biomarkers (IL-6, IL-8, IL-10 and ratio of IL-6 to IL-10), patients’ characteristics (age) and chest CT images (consolidation, emphysema and residual healthy lung parenchyma) have been reported to predict the prognosis of COVID-19 patients [8, 36]. In addition, the innovative method of machine learning is also used to precisely evaluate the COVID-19 pneumonia [37].

In this study, based on the clinical information of hospitalized adult COVID-19 patients, we identified CEA as a prognostic indicator for COVID-19 patients independently. Additionally, the prognostic nomogram including CEA was also constructed with a good applicability (AUC = 0.776). CEA, initially considered as an oncofetal protein, is an epithelial cell glycoprotein with a molecular mass of 180–200 kDa. At present, CEA is viewed as a normal epithelial molecule and its abnormal expression is generally found in tumors [38].

In COVID-19, we also found that CEACAM8 is highly expressed in the developing neutrophils/neutrophil progenitors, while CEACAM5 and CEACAM6 are highly expressed in type II pneumocyte. In humans, CEA and CEA subfamily members (CEACAMs) are cell surface heavily glycosylated proteins. In the bacterial or viral infection, CEA and CEACAM1 participate in the adherence of enteric bacteria to the apical membrane of colonic M cells in the human gut mucosa [39]. Besides, in the human respiratory tract, CEACAM1 and CEACAM5 increase the host susceptibility to bacterial infection upon viral challenge [40].

COVID-19 can lead to fatal comorbidities, especially acute respiratory distress syndrome (ARDS), which mainly caused by the injury to the alveolar epithelial cells [41]. And it has been reported that the major risk factors for severe COVID-19 are shared with IPF, namely increasing age, male sex and comorbidities such as hypertension and diabetes [42]. Due to the close correlation between CEA and ALI/IPF, we initially speculated that the poor prognosis of COVID-19 patients mediated by CEA might be related to ALI and IPF pathophysiologically. Because there are no natural models for IPF and ALI, the use of animal models that reproduce key known features of the disease is warranted. Direct lung infection is the leading cause of ARDS/ALI and can be modeled in mice using live pathogens and sterile models of inflammation while the bleomycin mouse model has identified many of the molecular and cellular mechanisms recognized as being important in pathogenesis of IPF [43, 44]. Therefore to validate this hypothesis, scRNA-seq data of ALI mouse lungs and IPF mouse lungs were also downloaded to evaluate the distribution and expression of CRGs, key receptor–ligand pair of cellular communication and potential downstream pathways [26–30]. The cross talk via CEACAM8-CEACAM6 was found between developing neutrophils and type II pneumocyte in COVID-19 but not ALI and IPF suggesting that during COVID-19 infection process, the differentiated developing neutrophils might regulate some biological processes of type II pneumocyte. The previous study reported that some CEACAMs were shown to be receptors that facilitate entry of middle east respiratory syndrome coronavirus [45]. And CEACAM were involved in cell–cell recognition and modulate cellular processes that range from the shaping of tissue architecture and neovascularization to the regulation of insulin homeostasis and T-cell proliferation [46]. However, the role of CEACAM in COVID-19 remains hypothetical in ARDS pathophysiology.

In COVID-19, the developing neutrophils were found to have cross talk with type II pneumocyte via CEACAM8-CEACAM6. Generally, CEACAM can be engaged in cellular communication which may affect various signal transduction processes related to cell activation, differentiation and apoptosis [47, 48]. In this process, CEACAM8-CEACAM6 regulation network may promote the differentiation of developing neutrophils, which are the newly annotated cells in patients with ARDS and represent neutrophils at various developmental stages [16]. The developing neutrophils may further lead to COVID-19 progression and induce the ARDS. Besides, it also regulates the proliferation of type II pneumocyte, which highly expresses ACE2 and serves as the major infected cell type by SARS-CoV-2 [49].

CEA level has been reported to be correlated with severity of several lung disease [28, 50–52]. The close association between respiratory epithelial damage and the release of CEA in IPF has been validated by a study based on BALF and serum measurement of CEA [50]. Acute exacerbations of IPF is pathologically manifested as diffuse acute lung injury (DALI) on the basis of pulmonary interstitial fibrosis [28]. Since COVID-19 pneumonia belongs to interstitial pneumonia and IPF was the result of the final fibrosis of interstitial pneumonia, we initially speculated that the poor prognosis of COVID-19 patients mediated by CEA might be related to ALI and IPF pathophysiologically. However, abnormal expression of CRGs was not found in both scRNA-seq samples of ALI and IPF while no developing neutrophils were annotated. Thus, abnormal expressions of CRGs in COVID-19 patients were COVID-19-specific and not related to CEA involvement in ALI and IPF.

To the best of our knowledge, the present study was the first to systematically evaluate the prognostic roles of CEA in COVID-19 patients and implies the potential mechanism in BALF and PMBC. The results implied the potential for clinical application. However, several limitations were present in this study. First, the retrospective nature of the present study was a limitation compared with a prospective study. Secondly, the generalizability of the nomogram had not been validated externally by the multicenter data, nor had the potential mechanism of CRGs been verified by wet experiments. Third, the case number of scRNA-seq data of BALF from three patients with moderate COVID-19, six patients with severe or critical infection and three healthy controls was limited. Fourth, smokers could have increased CEA levels without any correlation with COVID-19. However, smoking status was unknown for more than 25% of the patients. Any conclusion seems therefore debatable. Due to the limited number of smoking patients in this study (11/300) and the large number of patients with unknown smoking status (79/300), the relationship between smoking status and CEA in COVID-19 patients needs to be further studied. Last but not least, the limitation of sample size may contribute to the major bias of this study. Subsequent studies should focus on clinical studies with larger sample sizes and higher levels of evidence and basic studies exploring the molecular mechanisms of key biomarkers.

## Conclusion

This study identifies prognostic roles of CEA in COVID-19 patients and implies the potential mechanism of CEACAM8-CEACAM6 in the progression of COVID-19 by regulating the cellular communication of developing neutrophils and type II pneumocyte. The abnormal expressions of CRGs in COVID-19 patients were COVID-19-specific and not related to CEA involvement in ALI and IPF.

## Supplementary Information

**Additional file 1**. **Table S1**: Results of Kaplan–Meier survival analysis of 17 laboratory values with more than 20% missing data.

**Additional file 2**. **Supplementary material 1**: Converged multivariate Cox regression model including 5 potential prognostic laboratory indicators with significance in univariate analysis (remove missing values). In the cohort where missing values were removed, only the regression models, respectively, including AST, ALT, ferritin, D-dimer and PLT were converged (due to the uneven distribution of some variables level and events number when removing missing values), suggesting that CEA was an independent prognostic factor in all multivariate models and both normal PLT (HR = 0.635, 95% CI (0.408 to 0.990), P = 0.045) and normal ferritin (HR = 0.094, 95% CI (0.010 to 0.860), P = 0.037) were also independent favorable factors compared abnormal levels.

**Additional file 3**. **Supplementary material 2**: Converged multivariate Cox regression model including 11 potential prognostic laboratory indicators with significance in univariate analysis (keep missing values). In the cohort keeping missing values, a total of 11 regression models were converged and CEA was an independent prognostic factor in all multivariate models. Additionally, patients with normal PLT (HR 0.624; 95% CI 0.406 to 0.960; P = 0.031), ferritin (HR 0.089; 95% CI 0.010 to 0.750; P = 0.026), IL − 6 (HR 0.494; 95% CI 0.264 to 0.930; P = 0.028) and myoglobin (HR 0.520; 95% CI 0.303 to 0.890; P = 0.017) had better OS than patients with abnormal levels of these laboratory indicators in the multivariate models.

**Additional file 4**: **Supplementary material 3**: Multivariate Cox regression model including smoking information (keep or remove missing values). As smokers or patients with specific malignancies could have increased CEA levels without any correlation with COVID-19, all patients with primary malignancy were excluded from the study. Furthermore, in order to identify the association between CEA levels and smoking, two subgroup Cox proportional hazard regression models including smoking status (keep or remove missing values) were constructed, suggesting that the CEA level (HR 0.547; 95% CI 0.318 to 0.940; P = 0.037) (remove missing values) (HR 0.620; 95% CI 0.384 to 0.990; P = 0.048) (keep missing values) was still an independent prognostic indicator for COVID-19 patients.

**Additional file 5**. **Figure S1**: Model diagnosis of prognostic nomogram. The calibration curve and time-related ROC suggested acceptable calibration and discrimination of the nomogram (A-B). Besides, the risk score (RS) was calculated by the formula generated by the multivariate Cox model. The scatter plot (C) and risk curve (D) of the model demonstrated the RS distribution based on risk score of each patient. And the residual distribution of the multivariate model was accessed by the residual plot (E). Additionally, the results of Kaplan–Meier analysis of 17 laboratory values with more than 20% missing data were illustrated by survival curves (F).

**Additional file 6**. **Figure S2**: Subgroup analysis between CEA and smoking status. The CRG expression levels were retrieved from the RNA-seq data of Lung Squamous Cell Carcinoma (LUSC) available from The Cancer Genome Atlas (TCGA). The results of rank-sum tests showed that CEA levels in both the serum of COVID-19 patients (A) and the tissues of lung cancer (B-C) were significantly higher in smokers than in non-smokers. Therefore, smoking status of patients should be considered when CEA was considered as a prognostic indicator.

**Additional file 7**. **Figure S3**: Kaplan–Meier curve evaluating the prognostic value of the mode of ventilation. To further evaluate the prognostic value of mode of ventilation, the Kaplan–Meier analysis was performed. The results suggested that mode of ventilation was significantly associated with the prognosis of COVID-19 patients (P < 0.001).

**Additional file 8**. **Figure S4**: Identification of cellular subpopulation in COVID-19 patients’ and healthy volunteers’ bronchoalveolar lavage fluid (BALF). scRNA-seq data of bronchoalveolar lavage fluid (BALF) from three patients with moderate COVID-19 (C141, C142 and C144), six patients with severe or critical infection (C143, C145, C146, C148, C149 and C152) and three healthy controls (C51, C52 and C100) (accession no. GSE145926) were download from the GEO database. A UAMP analysis was performed in 63,010 cells in BALF and clearly identified 20 clusters and 11 cell types (B cell, CD4 + T cell, CD8 + T cell, dendritic cell, macrophage, monocyte, natural killer cell, neutrophil, T cell: gammadelta, type I pneumocyte, type II pneumocyte) (A-B). The expression levels and expression percentages of the marker genes in each cell type were displayed in figure S5C and figure S5D, respectively (C-D).

**Additional file 9**. **Figure S5**: Cell cycle and cellphoneDB analysis in COVID-19 patients’ and healthy volunteers’ bronchoalveolar lavage fluid (BALF). Cell cycle analysis suggested that COVID-19 patients were more likely to have cells in the G2M and S stages (A-B). And cellphoneDB analysis illustrated that pneumocytes of COVID-19 patients communicated extensively with other immune cells through CRGs (C).

**Additional file 10**. **Figure S6**: Identification of cellular subpopulation in COVID-19 patients’ and healthy volunteers’ PBMCs. The UAMP analysis identified 18 clusters and 10 cell types (B cell, B cell Naïve, CD4 + T cell, CD8 + T cell, macrophage–monocyte, myelocyte, natural killer cell, neutrophil, plasma cell. platelets) (A-B). Besides, cell cycle analysis suggested that the developing neutrophils in COVID-19 patients’ PBMCs were all engaged in the G2M and S stages (C-D). And a more extensive cellular communication analysis performed by iTALK algorithm further illustrated mechanisms between the developing neutrophils and the other PBMCs (E–F)

**Additional file 11**. **Figure S7**: Subgroup analysis for all neutrophils in COVID-19 patients’ and healthy volunteers’ PBMCs. All neutrophils were extracted separately and re-analyzed for dimensionality reduction. The UAMP analysis identified two cell types including canonical neutrophils and developing neutrophils (A-B). A significant increase in the number of developing neutrophils was found in COVID-19 while CEACAM1, CEACAM6 and CEACAM8 were also significantly co-localized developing neutrophils (C-D).

## Data Availability

The datasets generated and/or analyzed during the current study are available in the Supplementary Material, Gene Expression Omnibus (GEO) (Accession no. GSE145926, GSE150728, GSE134383) and Single Cell Expression Atlas (Accession no. E-HCAD-14).
