# Supplementary material for: The predicting roles of carcinoembryonic antigen and its underlying mechanism in the progression of coronavirus disease 2019
Source: Crit Care. 2021 Jul 3;25:234. doi: 10.1186/s13054-021-03661-y (PMC8254455; doi:10.1186/s13054-021-03661-y)
Supplement: Supplementary file 1 — Additional file 1. Table S1: Results of Kaplan–Meier survival analysis of 17 laboratory values with more than 20% missing data. [file 13054_2021_3661_MOESM1_ESM.docx]

Table S1 The results of Kaplan-Meier survival analysis of 17 lab values with more than 20% missing data

| Variables | Range of normal value | Log-rank P value |
| --- | --- | --- |
| ALT (Alanine aminotransferase) | 5-40u/l | 0.028 |
| AST (Aspartate aminotransferase) | 13-35u/l | < 0.001 |
| eGFR (estimated Glomerular filtration rate) | >90ml/min1.73m2 | 0.138 |
| Cr (creatinine) | 41-81umol/l | 0.728 |
| PLT (Platelet) | 125-350×10^9/l | 0.004 |
| PCT (Procalcitonin) | 0-0.5ng/ml | < 0.001 |
| SAA (Serum amyloid A) | 0-10mg/l | 0.090 |
| HBDH (α-hydroxybutyrate dehydrogenase) | 72-182u/l | 0.010 |
| LDH (Lactate dehydrogenase) | 120-250u/l | < 0.001 |
| Ferritin | 4.63-204ng/ml | 0.004 |
| IL-6 (Interleukin 6) | 0-7pg/ml | 0.009 |
| D-Dimer | 0-1.5ug/ml | < 0.001 |
| Fibrinogen | 2-4g/l | 0.104 |
| ESR (Erythrocyte sedimentation rate) | <20mm/60min | 0.668 |
| Myoglobin | 0-149pg/ml | < 0.001 |
| HsTNT (High sensitivity troponin) | 0-28pg/ml | < 0.001 |
| BNP (Brain natriuretic peptide) | 0-100pg/ml | 0.449 |
