# Supplementary material for: The predicting roles of carcinoembryonic antigen and its underlying mechanism in the progression of coronavirus disease 2019
Source: Crit Care. 2021 Jul 3;25:234. doi: 10.1186/s13054-021-03661-y (PMC8254455; doi:10.1186/s13054-021-03661-y)
Supplement: Supplementary file 2 — Additional file 2. Supplementary material 1: Converged multivariate Cox regression model including 5 potential prognostic laboratory indicators with significance in univariate analysis (remove missing values). In the cohort where missing values were removed, only the regression models, respectively, including AST, ALT, ferritin, D-dimer and PLT were converged (due to the uneven distribution of some variables level and events number when removing missing values), suggesting that CEA was an independent prognostic factor in all multivariate models and both normal PLT (HR = 0.635, 95% CI (0.408 to 0.990), P = 0.045) and normal ferritin (HR = 0.094, 95% CI (0.010 to 0.860), P = 0.037) were also independent favorable factors compared abnormal levels. [file 13054_2021_3661_MOESM2_ESM.pdf]

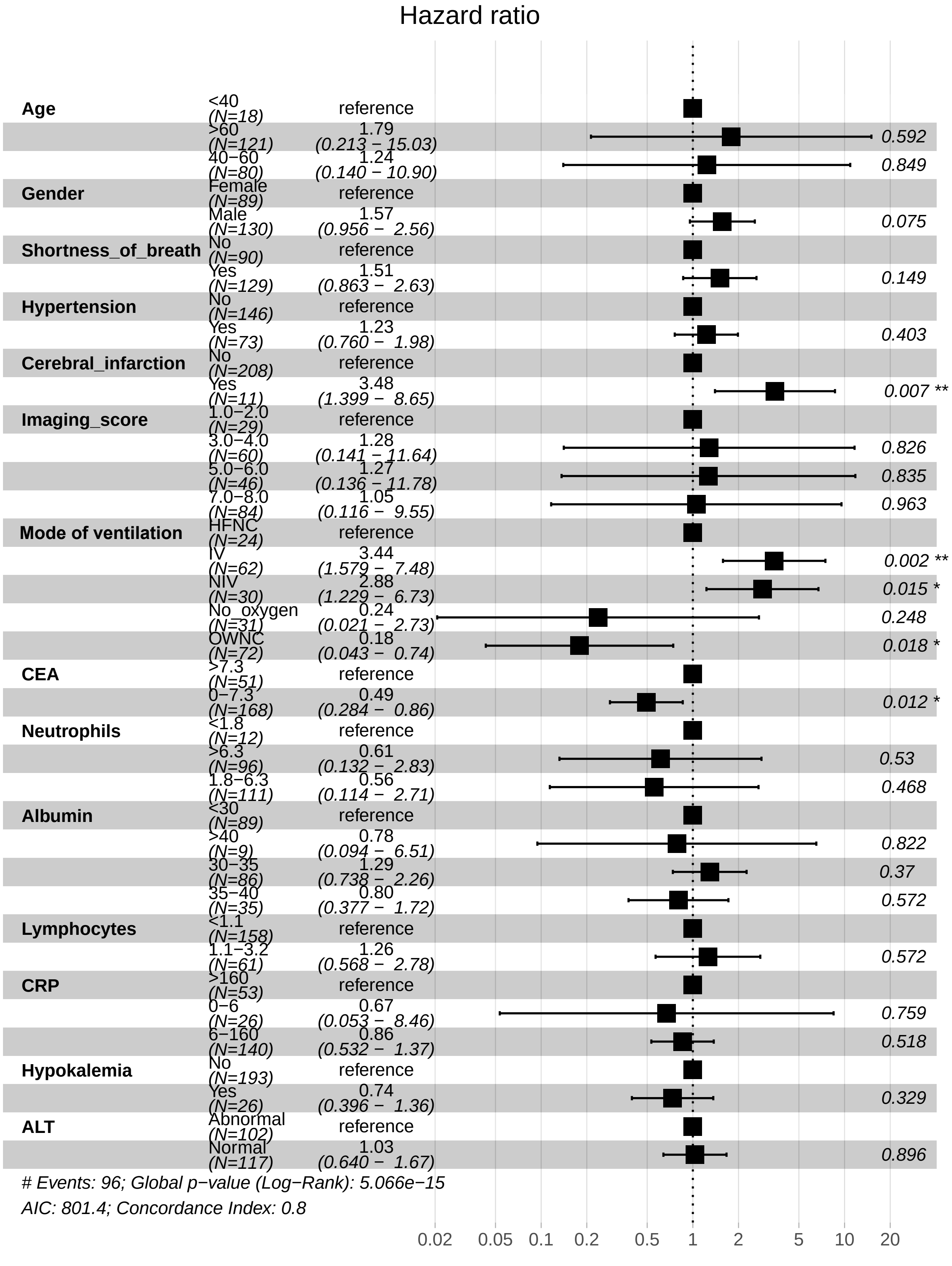

Hazard ratio
Age
<40 (N=18) reference
>60 (N=121) 1.79 (0.213 − 15.03) 0.592
40−60 (N=80) 1.24 (0.140 − 10.90) 0.849
Gender
Female (N=89) reference
Male (N=130) 1.57 (0.956 − 2.56) 0.075
Shortness_of_breath
No (N=90) reference
Yes (N=129) 1.51 (0.863 − 2.63) 0.149
Hypertension
No (N=146) reference
Yes (N=73) 1.23 (0.760 − 1.98) 0.403
Cerebral_infarction
No (N=208) reference
Yes (N=11) 3.48 (1.399 − 8.65) 0.007 **
Imaging_score
1.0−2.0 (N=29) reference
3.0−4.0 (N=60) 1.28 (0.141 − 11.64) 0.826
5.0−6.0 (N=46) 1.27 (0.136 − 11.78) 0.835
7.0−8.0 (N=84) 1.05 (0.116 − 9.55) 0.963
Mode of ventilation
HFNC (N=24) reference
IV (N=62) 3.44 (1.579 − 7.48) 0.002 **
NIV (N=30) 2.88 (1.229 − 6.73) 0.015 *
No_oxygen (N=31) 0.24 (0.021 − 2.73) 0.248
OWNC (N=72) 0.18 (0.043 − 0.74) 0.018 *
CEA
>7.3 (N=51) reference
0−7.3 (N=168) 0.49 (0.284 − 0.86) 0.012 *
Neutrophils
<1.8 (N=12) reference
>6.3 (N=96) 0.61 (0.132 − 2.83) 0.53
1.8−6.3 (N=111) 0.56 (0.114 − 2.71) 0.468
Albumin
<30 (N=89) reference
>40 (N=9) 0.78 (0.094 − 6.51) 0.822
30−35 (N=86) 1.29 (0.738 − 2.26) 0.37
35−40 (N=35) 0.80 (0.377 − 1.72) 0.572
Lymphocytes
<1.1 (N=158) reference
1.1−3.2 (N=61) 1.26 (0.568 − 2.78) 0.572
CRP
>160 (N=53) reference
0−6 (N=26) 0.67 (0.053 − 8.46) 0.759
6−160 (N=140) 0.86 (0.532 − 1.37) 0.518
Hypokalemia
No (N=193) reference
Yes (N=26) 0.74 (0.396 − 1.36) 0.329
ALT
Abnormal (N=102) reference
Normal (N=117) 1.03 (0.640 − 1.67) 0.896
Events: 96; Global p−value (Log−Rank): 5.066e−15
AIC: 801.4; Concordance Index: 0.8
0.02 0.05 0.1 0.2 0.5 1 2 5 10 20

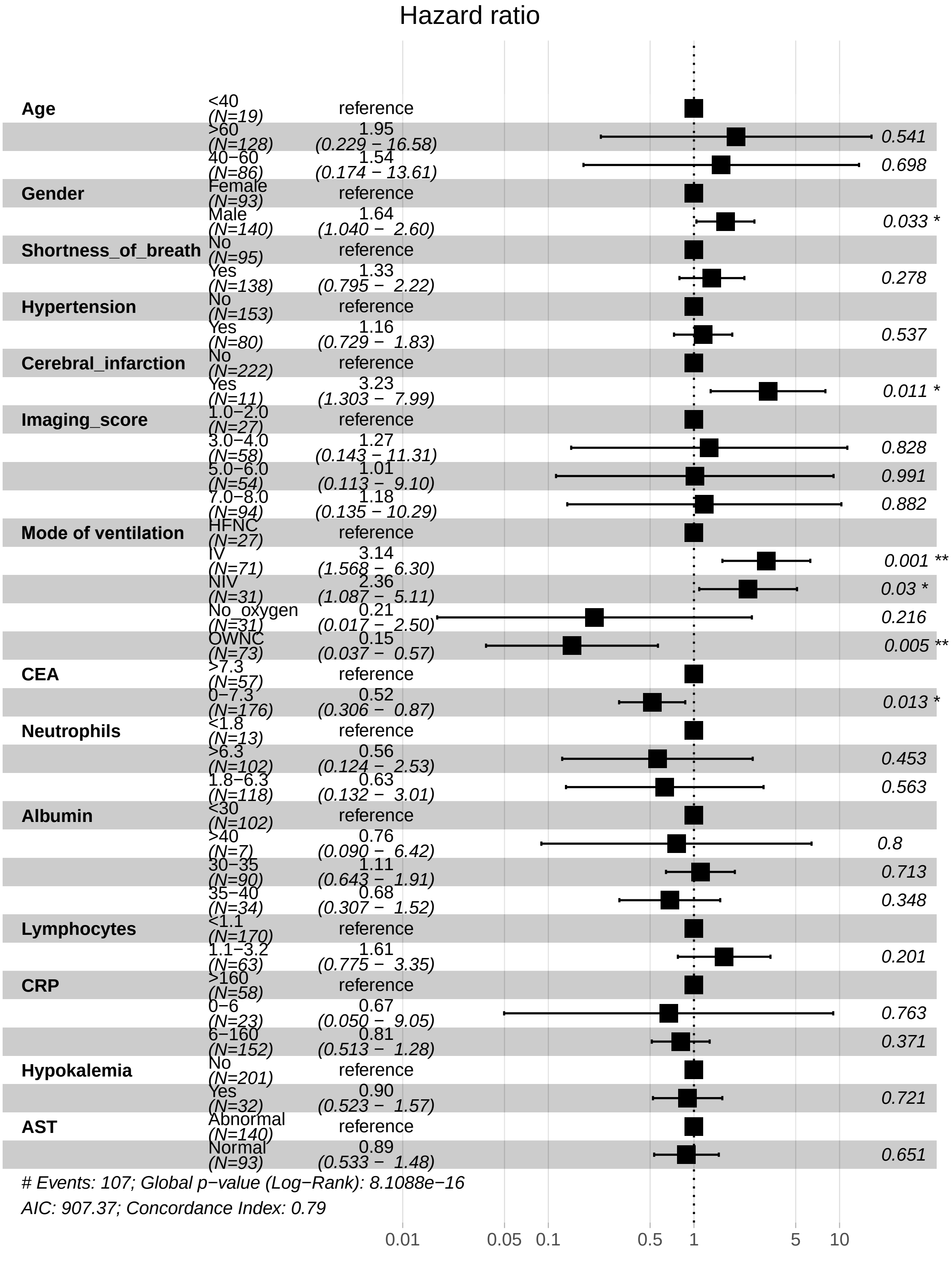
Hazard ratio
Age
<40 (N=19) reference
>60 (N=128) 1.95 (0.229 − 16.58) 0.541
40−60 (N=86) 1.54 (0.174 − 13.61) 0.698
Gender
Female (N=93) reference
Male (N=140) 1.64 (1.040 − 2.60) 0.033 *
Shortness_of_breath
No (N=95) reference
Yes (N=138) 1.33 (0.795 − 2.22) 0.278
Hypertension
No (N=153) reference
Yes (N=80) 1.16 (0.729 − 1.83) 0.537
Cerebral_infarction
No (N=222) reference
Yes (N=11) 3.23 (1.303 − 7.99) 0.011 *
Imaging_score
1.0−2.0 (N=27) reference
3.0−4.0 (N=58) 1.27 (0.143 − 11.31) 0.828
5.0−6.0 (N=54) 1.01 (0.113 − 9.10) 0.991
7.0−8.0 (N=94) 1.18 (0.135 − 10.29) 0.882
Mode of ventilation
HFNC (N=27) reference
IV (N=71) 3.14 (1.568 − 6.30) 0.001 **
NIV (N=31) 2.36 (1.087 − 5.11) 0.03 *
No_oxygen (N=31) 0.21 (0.017 − 2.50) 0.216
OWNC (N=73) 0.15 (0.037 − 0.57) 0.005 **
CEA
>7.3 (N=57) reference
0−7.3 (N=176) 0.52 (0.306 − 0.87) 0.013 *
Neutrophils
<1.8 (N=13) reference
>6.3 (N=102) 0.56 (0.124 − 2.53) 0.453
1.8−6.3 (N=118) 0.63 (0.132 − 3.01) 0.563
Albumin
<30 (N=102) reference
>40 (N=7) 0.76 (0.090 − 6.42) 0.8
30−35 (N=90) 1.11 (0.643 − 1.91) 0.713
35−40 (N=34) 0.68 (0.307 − 1.52) 0.348
Lymphocytes
<1.1 (N=170) reference
1.1−3.2 (N=63) 1.61 (0.775 − 3.35) 0.201
CRP
>160 (N=58) reference
0−6 (N=23) 0.67 (0.050 − 9.05) 0.763
6−160 (N=152) 0.81 (0.513 − 1.28) 0.371
Hypokalemia
No (N=201) reference
Yes (N=32) 0.90 (0.523 − 1.57) 0.721
AST
Abnormal (N=140) reference
Normal (N=93) 0.89 (0.533 − 1.48) 0.651
Events: 107; Global p−value (Log−Rank): 8.1088e−16
AIC: 907.37; Concordance Index: 0.79
0.01
0.05
0.1
0.5
1
5
10

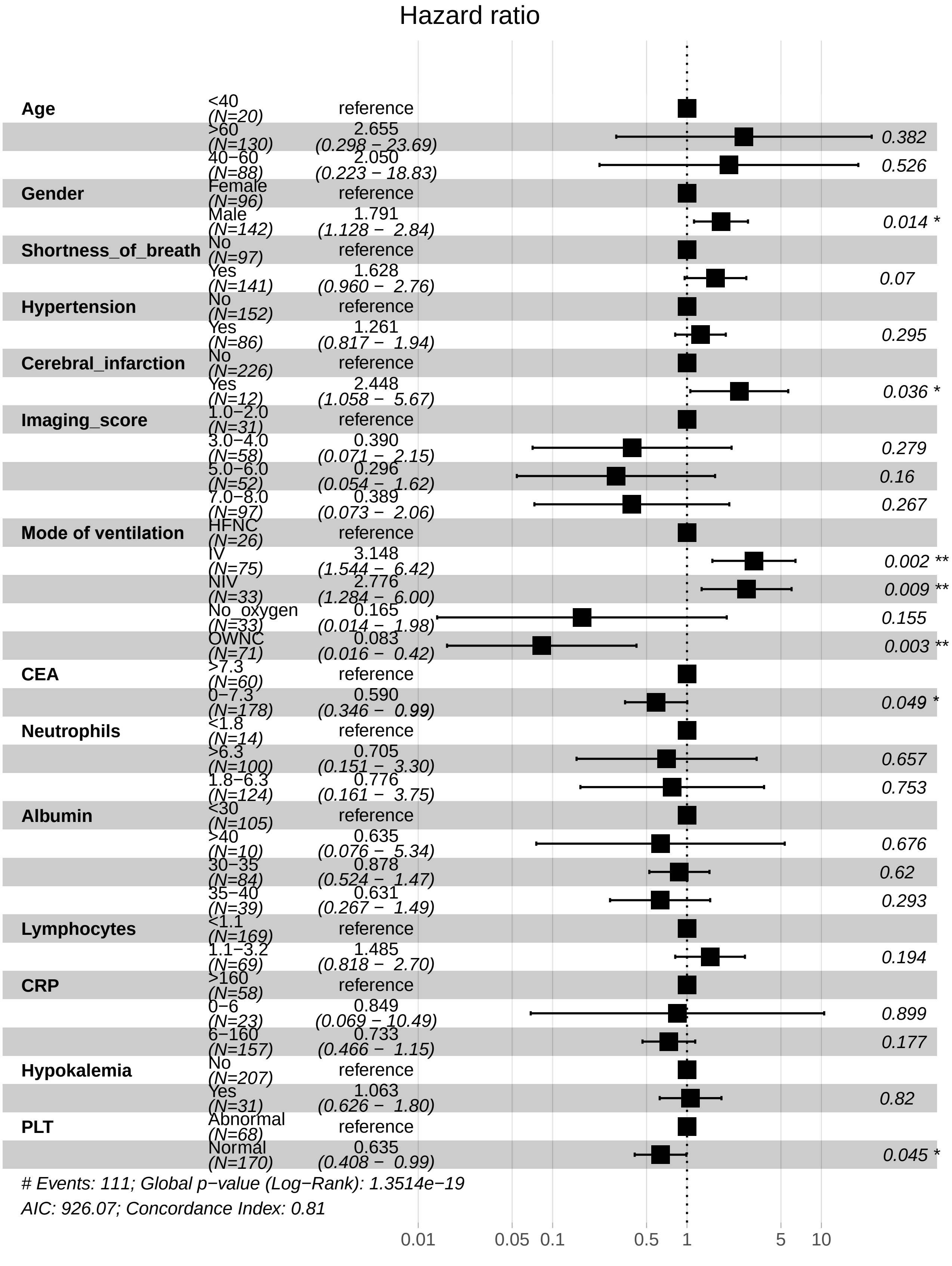

Hazard ratio
Age
<40 (N=20) reference
>60 (N=130) 2.655 (0.298 − 23.69) 0.382
40−60 (N=88) 2.050 (0.223 − 18.83) 0.526
Gender
Female (N=96) reference
Male (N=142) 1.791 (1.128 − 2.84) 0.014 *
Shortness_of_breath
No (N=97) reference
Yes (N=141) 1.628 (0.960 − 2.76) 0.07
Hypertension
No (N=152) reference
Yes (N=86) 1.261 (0.817 − 1.94) 0.295
Cerebral_infarction
No (N=226) reference
Yes (N=12) 2.448 (1.058 − 5.67) 0.036 *
Imaging_score
1.0−2.0 (N=31) reference
3.0−4.0 (N=58) 0.390 (0.071 − 2.15) 0.279
5.0−6.0 (N=52) 0.296 (0.054 − 1.62) 0.16
7.0−8.0 (N=97) 0.389 (0.073 − 2.06) 0.267
Mode of ventilation
HFNC (N=26) reference
IV (N=75) 3.148 (1.544 − 6.42) 0.002 **
NIV (N=33) 2.776 (1.284 − 6.00) 0.009 **
No_oxygen (N=33) 0.165 (0.014 − 1.98) 0.155
OWNC (N=71) 0.083 (0.016 − 0.42) 0.003 **
CEA
>7.3 (N=60) reference
0−7.3 (N=178) 0.590 (0.346 − 0.99) 0.049 *
Neutrophils
<1.8 (N=14) reference
>6.3 (N=100) 0.705 (0.151 − 3.30) 0.657
1.8−6.3 (N=124) 0.776 (0.161 − 3.75) 0.753
Albumin
<30 (N=105) reference
>40 (N=10) 0.635 (0.076 − 5.34) 0.676
30−35 (N=84) 0.878 (0.524 − 1.47) 0.62
35−40 (N=39) 0.631 (0.267 − 1.49) 0.293
Lymphocytes
<1.1 (N=169) reference
1.1−3.2 (N=69) 1.485 (0.818 − 2.70) 0.194
CRP
>160 (N=58) reference
0−6 (N=23) 0.849 (0.069 − 10.49) 0.899
6−160 (N=157) 0.733 (0.466 − 1.15) 0.177
Hypokalemia
No (N=207) reference
Yes (N=31) 1.063 (0.626 − 1.80) 0.82
PLT
Abnormal (N=68) reference
Normal (N=170) 0.635 (0.408 − 0.99) 0.045 *
Events: 111; Global p−value (Log−Rank): 1.3514e−19
AIC: 926.07; Concordance Index: 0.81
0.01
0.05
0.1
0.5
1
5
10

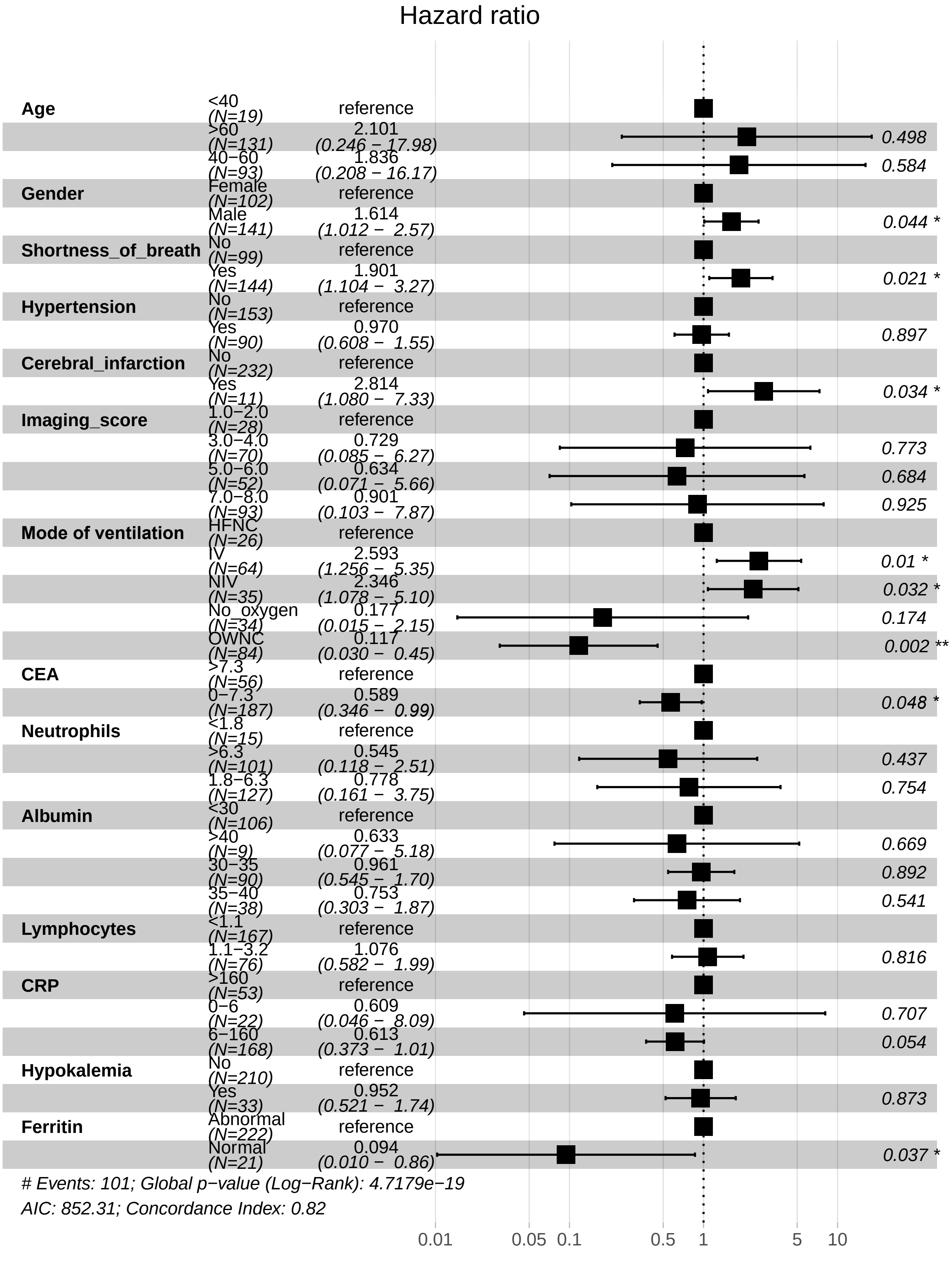
Hazard ratio
Age
<40 (N=19) reference
>60 (N=131) 2.101 (0.246 − 17.98) 0.498
40−60 (N=93) 1.836 (0.208 − 16.17) 0.584
Gender
Female (N=102) reference
Male (N=141) 1.614 (1.012 − 2.57) 0.044 *
Shortness_of_breath
No (N=99) reference
Yes (N=144) 1.901 (1.104 − 3.27) 0.021 *
Hypertension
No (N=153) reference
Yes (N=90) 0.970 (0.608 − 1.55) 0.897
Cerebral_infarction
No (N=232) reference
Yes (N=11) 2.814 (1.080 − 7.33) 0.034 *
Imaging_score
1.0−2.0 (N=28) reference
3.0−4.0 (N=70) 0.729 (0.085 − 6.27) 0.773
5.0−6.0 (N=52) 0.634 (0.071 − 5.66) 0.684
7.0−8.0 (N=93) 0.901 (0.103 − 7.87) 0.925
Mode of ventilation
HFNC (N=26) reference
IV (N=64) 2.593 (1.256 − 5.35) 0.01 *
NIV (N=35) 2.346 (1.078 − 5.10) 0.032 *
No_oxygen (N=34) 0.177 (0.015 − 2.15) 0.174
OWNC (N=84) 0.117 (0.030 − 0.45) 0.002 **
CEA
>7.3 (N=56) reference
0−7.3 (N=187) 0.589 (0.346 − 0.99) 0.048 *
Neutrophils
<1.8 (N=15) reference
>6.3 (N=101) 0.545 (0.118 − 2.51) 0.437
1.8−6.3 (N=127) 0.778 (0.161 − 3.75) 0.754
Albumin
<30 (N=106) reference
>40 (N=9) 0.633 (0.077 − 5.18) 0.669
30−35 (N=90) 0.961 (0.545 − 1.70) 0.892
35−40 (N=38) 0.753 (0.303 − 1.87) 0.541
Lymphocytes
<1.1 (N=167) reference
1.1−3.2 (N=76) 1.076 (0.582 − 1.99) 0.816
CRP
>160 (N=53) reference
0−6 (N=22) 0.609 (0.046 − 8.09) 0.707
6−160 (N=168) 0.613 (0.373 − 1.01) 0.054
Hypokalemia
No (N=210) reference
Yes (N=33) 0.952 (0.521 − 1.74) 0.873
Ferritin
Abnormal (N=222) reference
Normal (N=21) 0.094 (0.010 − 0.86) 0.037 *
Events: 101; Global p−value (Log−Rank): 4.7179e−19
AIC: 852.31; Concordance Index: 0.82
0.01
0.05
0.1
0.5
1
5
10

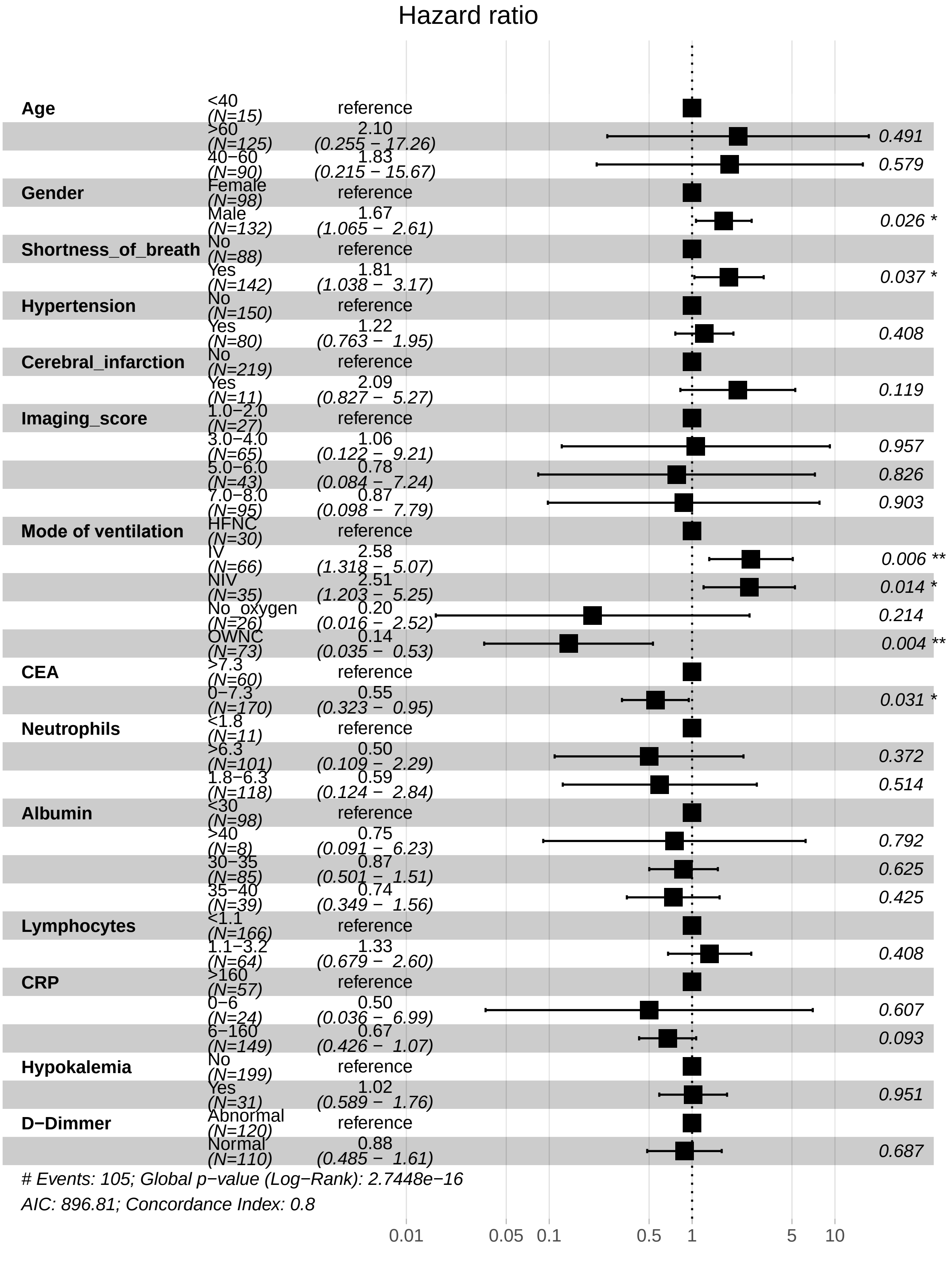

Hazard ratio
Age
<40 (N=15) reference
>60 (N=125) 2.10 (0.255 − 17.26) 0.491
40−60 (N=90) 1.83 (0.215 − 15.67) 0.579
Gender
Female (N=98) reference
Male (N=132) 1.67 (1.065 − 2.61) 0.026 *
Shortness_of_breath
No (N=88) reference
Yes (N=142) 1.81 (1.038 − 3.17) 0.037 *
Hypertension
No (N=150) reference
Yes (N=80) 1.22 (0.763 − 1.95) 0.408
Cerebral_infarction
No (N=219) reference
Yes (N=11) 2.09 (0.827 − 5.27) 0.119
Imaging_score
1.0−2.0 (N=27) reference
3.0−4.0 (N=65) 1.06 (0.122 − 9.21) 0.957
5.0−6.0 (N=43) 0.78 (0.084 − 7.24) 0.826
7.0−8.0 (N=95) 0.87 (0.098 − 7.79) 0.903
Mode of ventilation
HFNC (N=30) reference
IV (N=66) 2.58 (1.318 − 5.07) 0.006 **
NIV (N=35) 2.51 (1.203 − 5.25) 0.014 *
No_oxygen (N=26) 0.20 (0.016 − 2.52) 0.214
OWNC (N=73) 0.14 (0.035 − 0.53) 0.004 **
CEA
>7.3 (N=60) reference
0−7.3 (N=170) 0.55 (0.323 − 0.95) 0.031 *
Neutrophils
<1.8 (N=11) reference
>6.3 (N=101) 0.50 (0.109 − 2.29) 0.372
1.8−6.3 (N=118) 0.59 (0.124 − 2.84) 0.514
Albumin
<30 (N=98) reference
>40 (N=8) 0.75 (0.091 − 6.23) 0.792
30−35 (N=85) 0.87 (0.501 − 1.51) 0.625
35−40 (N=39) 0.74 (0.349 − 1.56) 0.425
Lymphocytes
<1.1 (N=166) reference
1.1−3.2 (N=64) 1.33 (0.679 − 2.60) 0.408
CRP
>160 (N=57) reference
0−6 (N=24) 0.50 (0.036 − 6.99) 0.607
6−160 (N=149) 0.67 (0.426 − 1.07) 0.093
Hypokalemia
No (N=199) reference
Yes (N=31) 1.02 (0.589 − 1.76) 0.951
D−Dimmer
Abnormal (N=120) reference
Normal (N=110) 0.88 (0.485 − 1.61) 0.687
Events: 105; Global p−value (Log−Rank): 2.7448e−16
AIC: 896.81; Concordance Index: 0.8
0.01
0.05
0.1
0.5
1
5
10
