# Supplementary material for: The predicting roles of carcinoembryonic antigen and its underlying mechanism in the progression of coronavirus disease 2019
Source: Crit Care. 2021 Jul 3;25:234. doi: 10.1186/s13054-021-03661-y (PMC8254455; doi:10.1186/s13054-021-03661-y)
Supplement: Supplementary file 3 — Additional file 3. Supplementary material 2: Converged multivariate Cox regression model including 11 potential prognostic laboratory indicators with significance in univariate analysis (keep missing values). In the cohort keeping missing values, a total of 11 regression models were converged and CEA was an independent prognostic factor in all multivariate models. Additionally, patients with normal PLT (HR 0.624; 95% CI 0.406 to 0.960; P = 0.031), ferritin (HR 0.089; 95% CI 0.010 to 0.750; P = 0.026), IL − 6 (HR 0.494; 95% CI 0.264 to 0.930; P = 0.028) and myoglobin (HR 0.520; 95% CI 0.303 to 0.890; P = 0.017) had better OS than patients with abnormal levels of these laboratory indicators in the multivariate models. [file 13054_2021_3661_MOESM3_ESM.pdf]

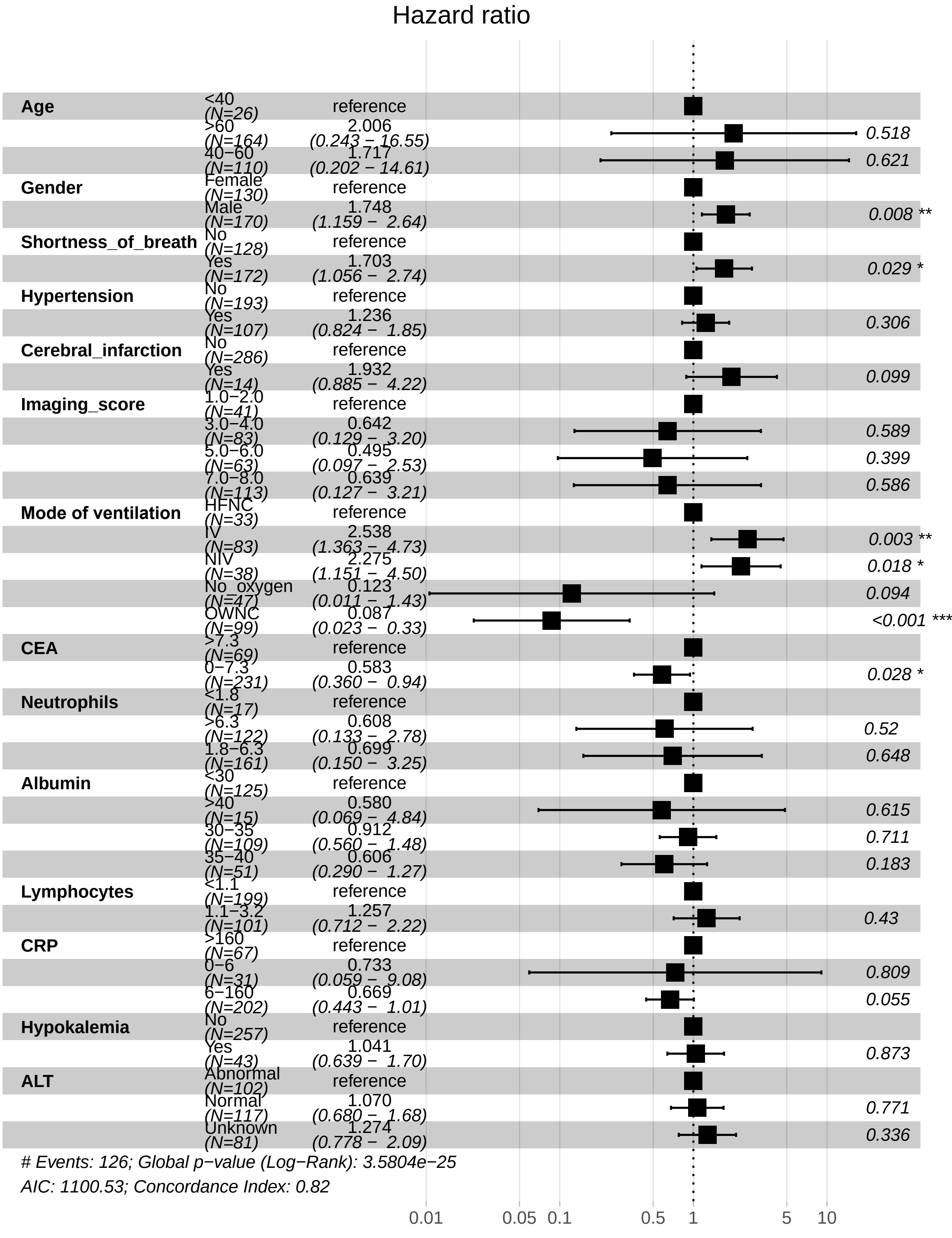

Hazard ratio
Age
<40 (N=26) reference
>60 (N=164) 2.006 (0.243 − 16.55) 0.518
40−60 (N=110) 1.717 (0.202 − 14.61) 0.621
Gender
Female (N=130) reference
Male (N=170) 1.748 (1.159 − 2.64) 0.008 **
Shortness_of_breath
No (N=128) reference
Yes (N=172) 1.703 (1.056 − 2.74) 0.029 *
Hypertension
No (N=193) reference
Yes (N=107) 1.236 (0.824 − 1.85) 0.306
Cerebral_infarction
No (N=286) reference
Yes (N=14) 1.932 (0.885 − 4.22) 0.099
Imaging_score
1.0−2.0 (N=41) reference
3.0−4.0 (N=83) 0.642 (0.129 − 3.20) 0.589
5.0−6.0 (N=63) 0.495 (0.097 − 2.53) 0.399
7.0−8.0 (N=113) 0.639 (0.127 − 3.21) 0.586
Mode of ventilation
HFNC (N=33) reference
IV (N=83) 2.538 (1.363 − 4.73) 0.003 **
NIV (N=38) 2.275 (1.151 − 4.50) 0.018 *
No_oxygen (N=47) 0.123 (0.011 − 1.43) 0.094
OWNC (N=99) 0.087 (0.023 − 0.33) <0.001 ***
CEA
>7.3 (N=69) reference
0−7.3 (N=231) 0.583 (0.360 − 0.94) 0.028 *
Neutrophils
<1.8 (N=17) reference
>6.3 (N=122) 0.608 (0.133 − 2.78) 0.52
1.8−6.3 (N=161) 0.699 (0.150 − 3.25) 0.648
Albumin
<30 (N=125) reference
>40 (N=15) 0.580 (0.069 − 4.84) 0.615
30−35 (N=109) 0.912 (0.560 − 1.48) 0.711
35−40 (N=51) 0.606 (0.290 − 1.27) 0.183
Lymphocytes
<1.1 (N=199) reference
1.1−3.2 (N=101) 1.257 (0.712 − 2.22) 0.43
CRP
>160 (N=67) reference
0−6 (N=31) 0.733 (0.059 − 9.08) 0.809
6−160 (N=202) 0.669 (0.443 − 1.01) 0.055
Hypokalemia
No (N=257) reference
Yes (N=43) 1.041 (0.639 − 1.70) 0.873
ALT
Abnormal (N=102) reference
Normal (N=117) 1.070 (0.680 − 1.68) 0.771
Unknown (N=81) 1.274 (0.778 − 2.09) 0.336
Events: 126; Global p−value (Log−Rank): 3.5804e−25
AIC: 1100.53; Concordance Index: 0.82
0.01 0.05 0.1 0.5 1 5 10

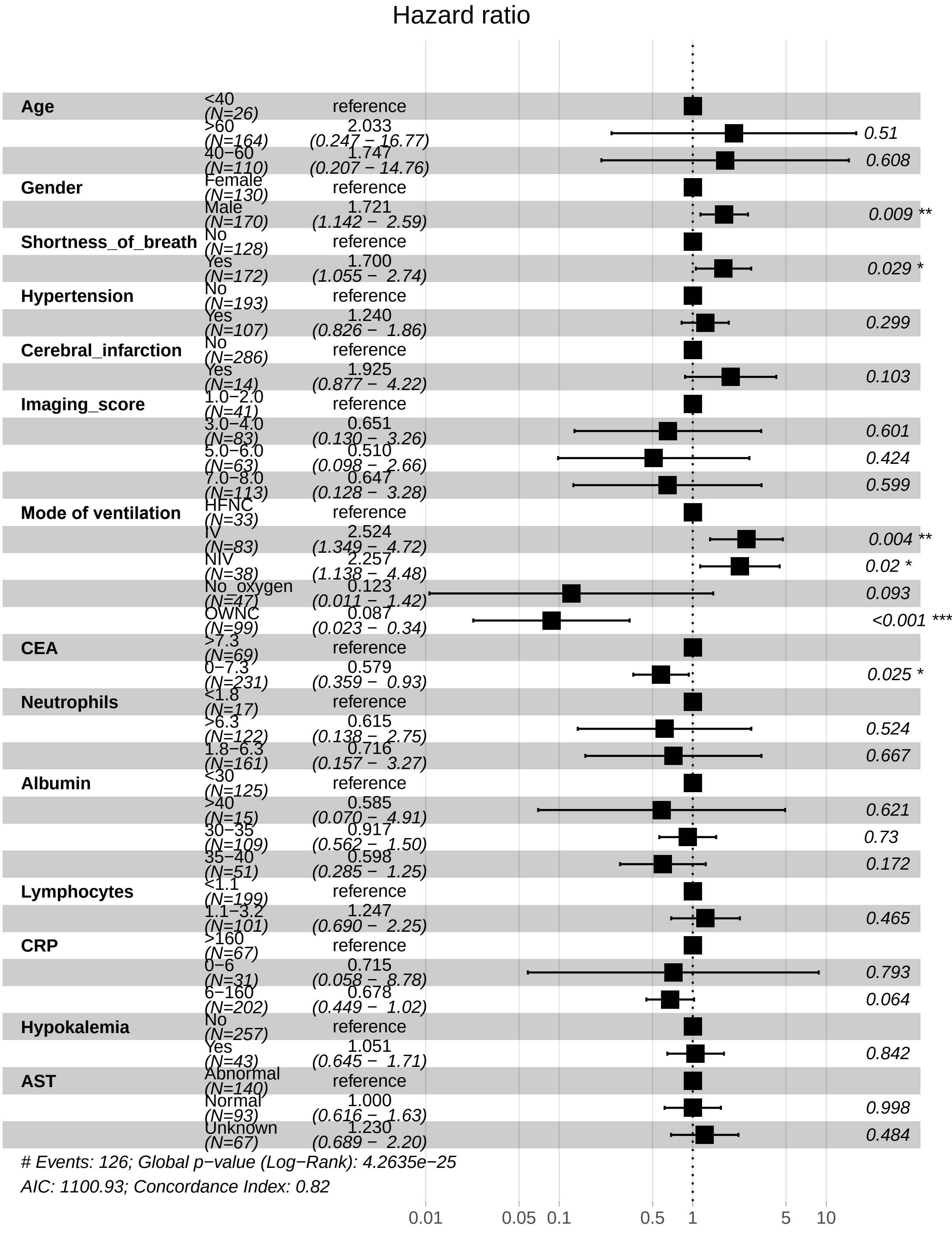

Hazard ratio
Age
<40 (N=26) reference
>60 (N=164) 2.033 (0.247 − 16.77) 0.51
40−60 (N=110) 1.747 (0.207 − 14.76) 0.608
Gender
Female (N=130) reference
Male (N=170) 1.721 (1.142 − 2.59) 0.009 **
Shortness_of_breath
No (N=128) reference
Yes (N=172) 1.700 (1.055 − 2.74) 0.029 *
Hypertension
No (N=193) reference
Yes (N=107) 1.240 (0.826 − 1.86) 0.299
Cerebral_infarction
No (N=286) reference
Yes (N=14) 1.925 (0.877 − 4.22) 0.103
Imaging_score
1.0−2.0 (N=41) reference
3.0−4.0 (N=83) 0.651 (0.130 − 3.26) 0.601
5.0−6.0 (N=63) 0.510 (0.098 − 2.66) 0.424
7.0−8.0 (N=113) 0.647 (0.128 − 3.28) 0.599
Mode of ventilation
HFNC (N=33) reference
IV (N=83) 2.524 (1.349 − 4.72) 0.004 **
NIV (N=38) 2.257 (1.138 − 4.48) 0.02 *
No_oxygen (N=47) 0.123 (0.011 − 1.42) 0.093
OWNC (N=99) 0.087 (0.023 − 0.34) <0.001 ***
CEA
>7.3 (N=69) reference
0−7.3 (N=231) 0.579 (0.359 − 0.93) 0.025 *
Neutrophils
<1.8 (N=17) reference
>6.3 (N=122) 0.615 (0.138 − 2.75) 0.524
1.8−6.3 (N=161) 0.716 (0.157 − 3.27) 0.667
Albumin
<30 (N=125) reference
>40 (N=15) 0.585 (0.070 − 4.91) 0.621
30−35 (N=109) 0.917 (0.562 − 1.50) 0.73
35−40 (N=51) 0.598 (0.285 − 1.25) 0.172
Lymphocytes
<1.1 (N=199) reference
1.1−3.2 (N=101) 1.247 (0.690 − 2.25) 0.465
CRP
>160 (N=67) reference
0−6 (N=31) 0.715 (0.058 − 8.78) 0.793
6−160 (N=202) 0.678 (0.449 − 1.02) 0.064
Hypokalemia
No (N=257) reference
Yes (N=43) 1.051 (0.645 − 1.71) 0.842
AST
Abnormal (N=140) reference
Normal (N=93) 1.000 (0.616 − 1.63) 0.998
Unknown (N=67) 1.230 (0.689 − 2.20) 0.484
Events: 126; Global p−value (Log−Rank): 4.2635e−25
AIC: 1100.93; Concordance Index: 0.82
0.01 0.05 0.1 0.5 1 5 10

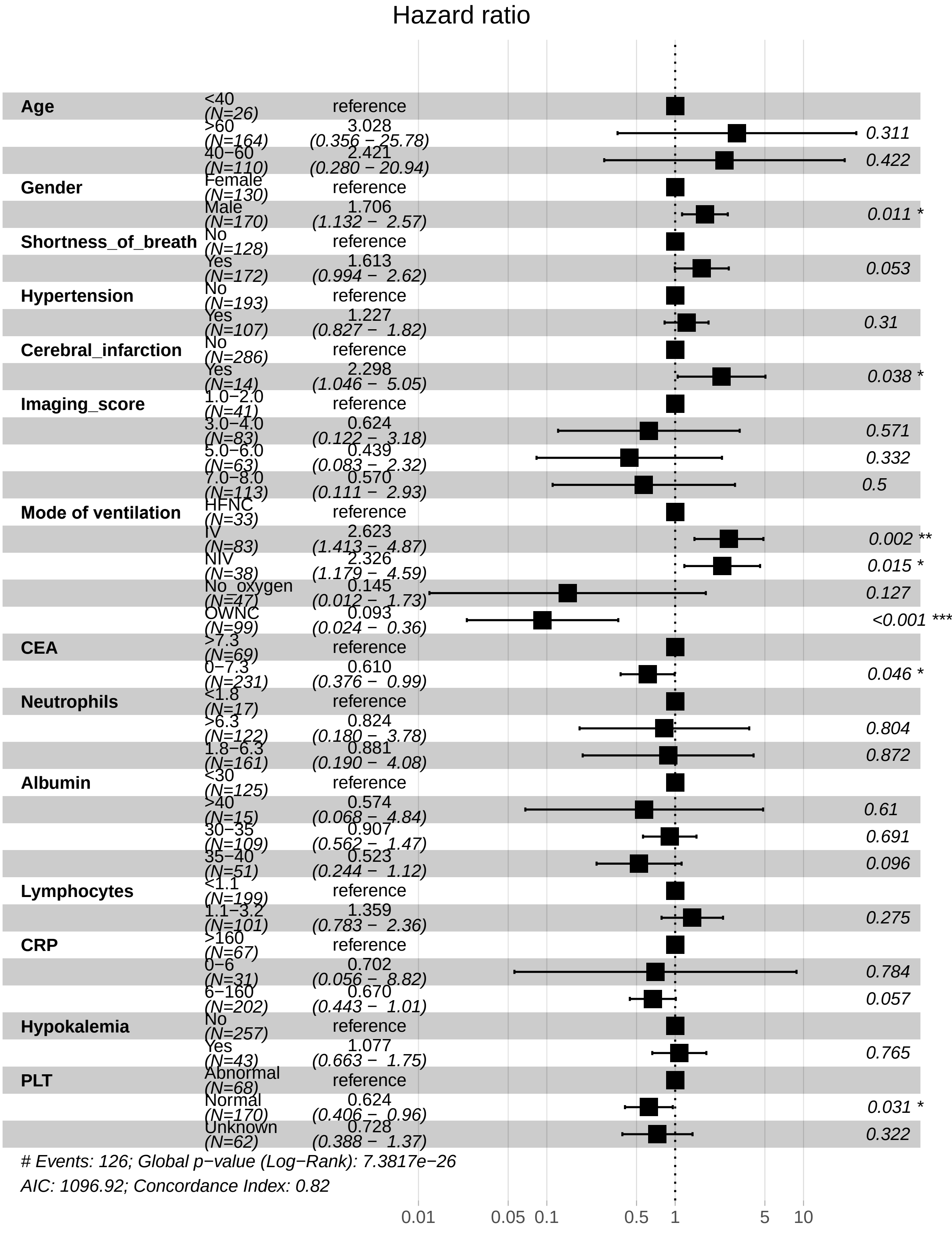

Hazard ratio
Age
<40 (N=26) reference
>60 (N=164) 3.028 (0.356 − 25.78) 0.311
40−60 (N=110) 2.421 (0.280 − 20.94) 0.422
Gender
Female (N=130) reference
Male (N=170) 1.706 (1.132 − 2.57) 0.011 *
Shortness_of_breath
No (N=128) reference
Yes (N=172) 1.613 (0.994 − 2.62) 0.053
Hypertension
No (N=193) reference
Yes (N=107) 1.227 (0.827 − 1.82) 0.31
Cerebral_infarction
No (N=286) reference
Yes (N=14) 2.298 (1.046 − 5.05) 0.038 *
Imaging_score
1.0−2.0 (N=41) reference
3.0−4.0 (N=83) 0.624 (0.122 − 3.18) 0.571
5.0−6.0 (N=63) 0.439 (0.083 − 2.32) 0.332
7.0−8.0 (N=113) 0.570 (0.111 − 2.93) 0.5
Mode of ventilation
HFNC (N=33) reference
IV (N=83) 2.623 (1.413 − 4.87) 0.002 **
NIV (N=38) 2.326 (1.179 − 4.59) 0.015 *
No_oxygen (N=47) 0.145 (0.012 − 1.73) 0.127
OWNC (N=99) 0.093 (0.024 − 0.36) <0.001 ***
CEA
>7.3 (N=69) reference
0−7.3 (N=231) 0.610 (0.376 − 0.99) 0.046 *
Neutrophils
<1.8 (N=17) reference
>6.3 (N=122) 0.824 (0.180 − 3.78) 0.804
1.8−6.3 (N=161) 0.881 (0.190 − 4.08) 0.872
Albumin
<30 (N=125) reference
>40 (N=15) 0.574 (0.068 − 4.84) 0.61
30−35 (N=109) 0.907 (0.562 − 1.47) 0.691
35−40 (N=51) 0.523 (0.244 − 1.12) 0.096
Lymphocytes
<1.1 (N=199) reference
1.1−3.2 (N=101) 1.359 (0.783 − 2.36) 0.275
CRP
>160 (N=67) reference
0−6 (N=31) 0.702 (0.056 − 8.82) 0.784
6−160 (N=202) 0.670 (0.443 − 1.01) 0.057
Hypokalemia
No (N=257) reference
Yes (N=43) 1.077 (0.663 − 1.75) 0.765
PLT
Abnormal (N=68) reference
Normal (N=170) 0.624 (0.406 − 0.96) 0.031 *
Unknown (N=62) 0.728 (0.388 − 1.37) 0.322
Events: 126; Global p−value (Log−Rank): 7.3817e−26
AIC: 1096.92; Concordance Index: 0.82
0.01 0.05 0.1 0.5 1 5 10

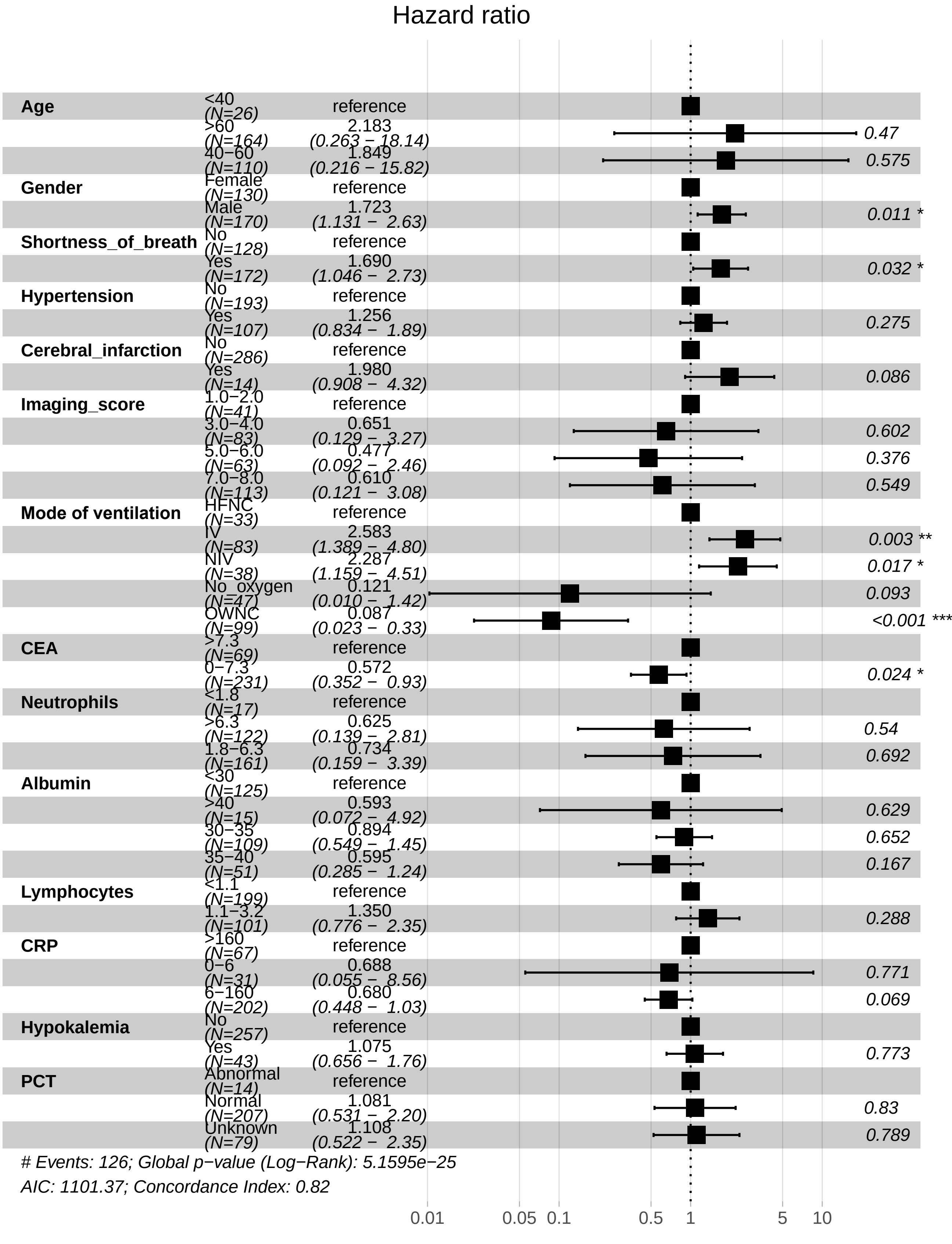

Hazard ratio
Age
<40 (N=26) reference
>60 (N=164) 2.183 (0.263 − 18.14) 0.47
40−60 (N=110) 1.849 (0.216 − 15.82) 0.575
Gender
Female (N=130) reference
Male (N=170) 1.723 (1.131 − 2.63) 0.011 *
Shortness_of_breath
No (N=128) reference
Yes (N=172) 1.690 (1.046 − 2.73) 0.032 *
Hypertension
No (N=193) reference
Yes (N=107) 1.256 (0.834 − 1.89) 0.275
Cerebral_infarction
No (N=286) reference
Yes (N=14) 1.980 (0.908 − 4.32) 0.086
Imaging_score
1.0−2.0 (N=41) reference
3.0−4.0 (N=83) 0.651 (0.129 − 3.27) 0.602
5.0−6.0 (N=63) 0.477 (0.092 − 2.46) 0.376
7.0−8.0 (N=113) 0.610 (0.121 − 3.08) 0.549
Mode of ventilation
HFNC (N=33) reference
IV (N=83) 2.583 (1.389 − 4.80) 0.003 **
NIV (N=38) 2.287 (1.159 − 4.51) 0.017 *
No_oxygen (N=47) 0.121 (0.010 − 1.42) 0.093
OWNC (N=99) 0.087 (0.023 − 0.33) <0.001 ***
CEA
>7.3 (N=69) reference
0−7.3 (N=231) 0.572 (0.352 − 0.93) 0.024 *
Neutrophils
<1.8 (N=17) reference
>6.3 (N=122) 0.625 (0.139 − 2.81) 0.54
1.8−6.3 (N=161) 0.734 (0.159 − 3.39) 0.692
Albumin
<30 (N=125) reference
>40 (N=15) 0.593 (0.072 − 4.92) 0.629
30−35 (N=109) 0.894 (0.549 − 1.45) 0.652
35−40 (N=51) 0.595 (0.285 − 1.24) 0.167
Lymphocytes
<1.1 (N=199) reference
1.1−3.2 (N=101) 1.350 (0.776 − 2.35) 0.288
CRP
>160 (N=67) reference
0−6 (N=31) 0.688 (0.055 − 8.56) 0.771
6−160 (N=202) 0.680 (0.448 − 1.03) 0.069
Hypokalemia
No (N=257) reference
Yes (N=43) 1.075 (0.656 − 1.76) 0.773
PCT
Abnormal (N=14) reference
Normal (N=207) 1.081 (0.531 − 2.20) 0.83
Unknown (N=79) 1.108 (0.522 − 2.35) 0.789
Events: 126; Global p−value (Log−Rank): 5.1595e−25
AIC: 1101.37; Concordance Index: 0.82
0.01 0.05 0.1 0.5 1 5 10

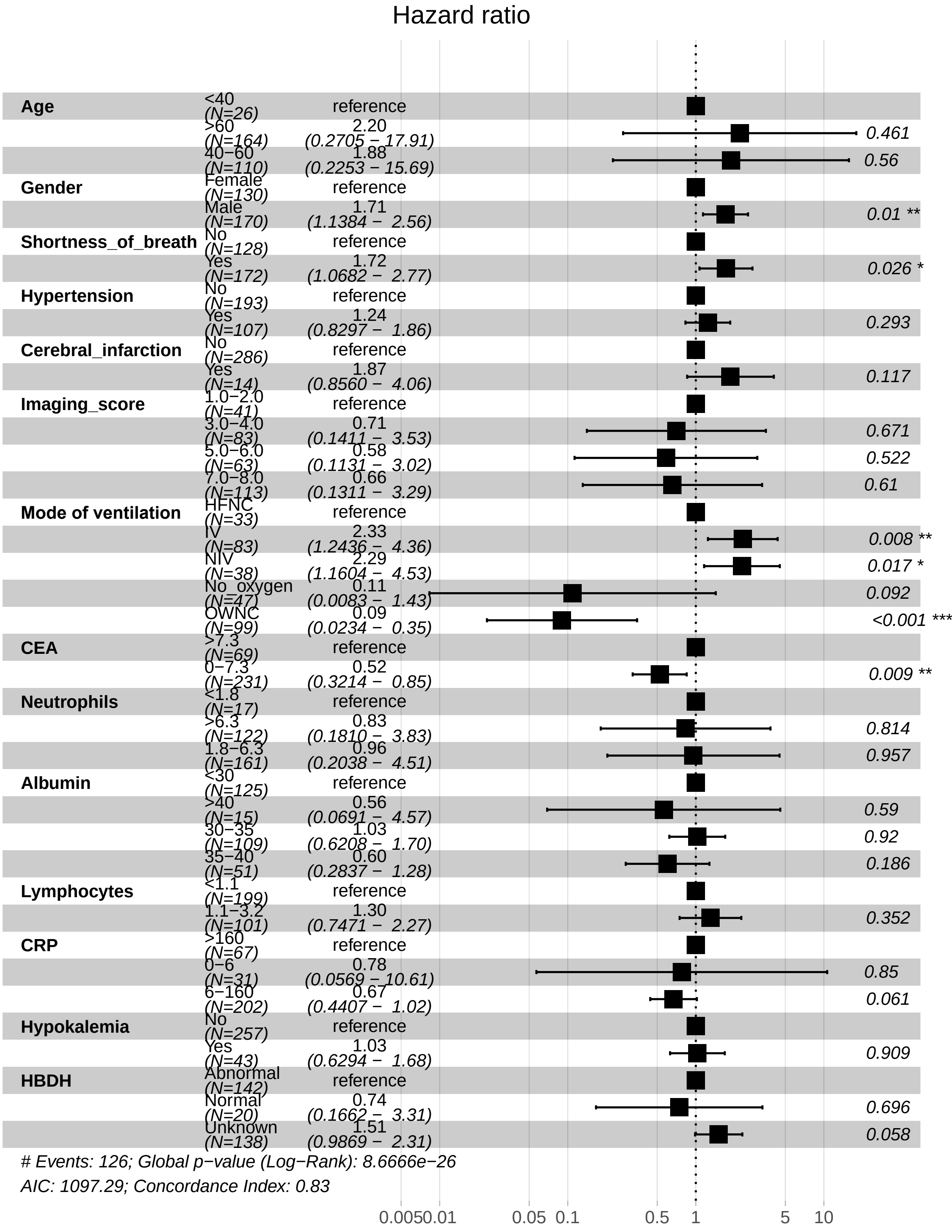
Hazard ratio
Age
<40 (N=26) reference
>60 (N=164) 2.20 (0.2705 − 17.91) 0.461
40−60 (N=110) 1.88 (0.2253 − 15.69) 0.56
Gender
Female (N=130) reference
Male (N=170) 1.71 (1.1384 − 2.56) 0.01 **
Shortness_of_breath
No (N=128) reference
Yes (N=172) 1.72 (1.0682 − 2.77) 0.026 *
Hypertension
No (N=193) reference
Yes (N=107) 1.24 (0.8297 − 1.86) 0.293
Cerebral_infarction
No (N=286) reference
Yes (N=14) 1.87 (0.8560 − 4.06) 0.117
Imaging_score
1.0−2.0 (N=41) reference
3.0−4.0 (N=83) 0.71 (0.1411 − 3.53) 0.671
5.0−6.0 (N=63) 0.58 (0.1131 − 3.02) 0.522
7.0−8.0 (N=113) 0.66 (0.1311 − 3.29) 0.61
Mode of ventilation
HFNC (N=33) reference
IV (N=83) 2.33 (1.2436 − 4.36) 0.008 **
NIV (N=38) 2.29 (1.1604 − 4.53) 0.017 *
No_oxygen (N=47) 0.11 (0.0083 − 1.43) 0.092
OWNC (N=99) 0.09 (0.0234 − 0.35) <0.001 ***
CEA
>7.3 (N=69) reference
0−7.3 (N=231) 0.52 (0.3214 − 0.85) 0.009 **
Neutrophils
<1.8 (N=17) reference
>6.3 (N=122) 0.83 (0.1810 − 3.83) 0.814
1.8−6.3 (N=161) 0.96 (0.2038 − 4.51) 0.957
Albumin
<30 (N=125) reference
>40 (N=15) 0.56 (0.0691 − 4.57) 0.59
30−35 (N=109) 1.03 (0.6208 − 1.70) 0.92
35−40 (N=51) 0.60 (0.2837 − 1.28) 0.186
Lymphocytes
<1.1 (N=199) reference
1.1−3.2 (N=101) 1.30 (0.7471 − 2.27) 0.352
CRP
>160 (N=67) reference
0−6 (N=31) 0.78 (0.0569 − 10.61) 0.85
6−160 (N=202) 0.67 (0.4407 − 1.02) 0.061
Hypokalemia
No (N=257) reference
Yes (N=43) 1.03 (0.6294 − 1.68) 0.909
HBDH
Abnormal (N=142) reference
Normal (N=20) 0.74 (0.1662 − 3.31) 0.696
Unknown (N=138) 1.51 (0.9869 − 2.31) 0.058
Events: 126; Global p−value (Log−Rank): 8.6666e−26
AIC: 1097.29; Concordance Index: 0.83
0.005 0.01 0.05 0.1 0.5 1 5 10

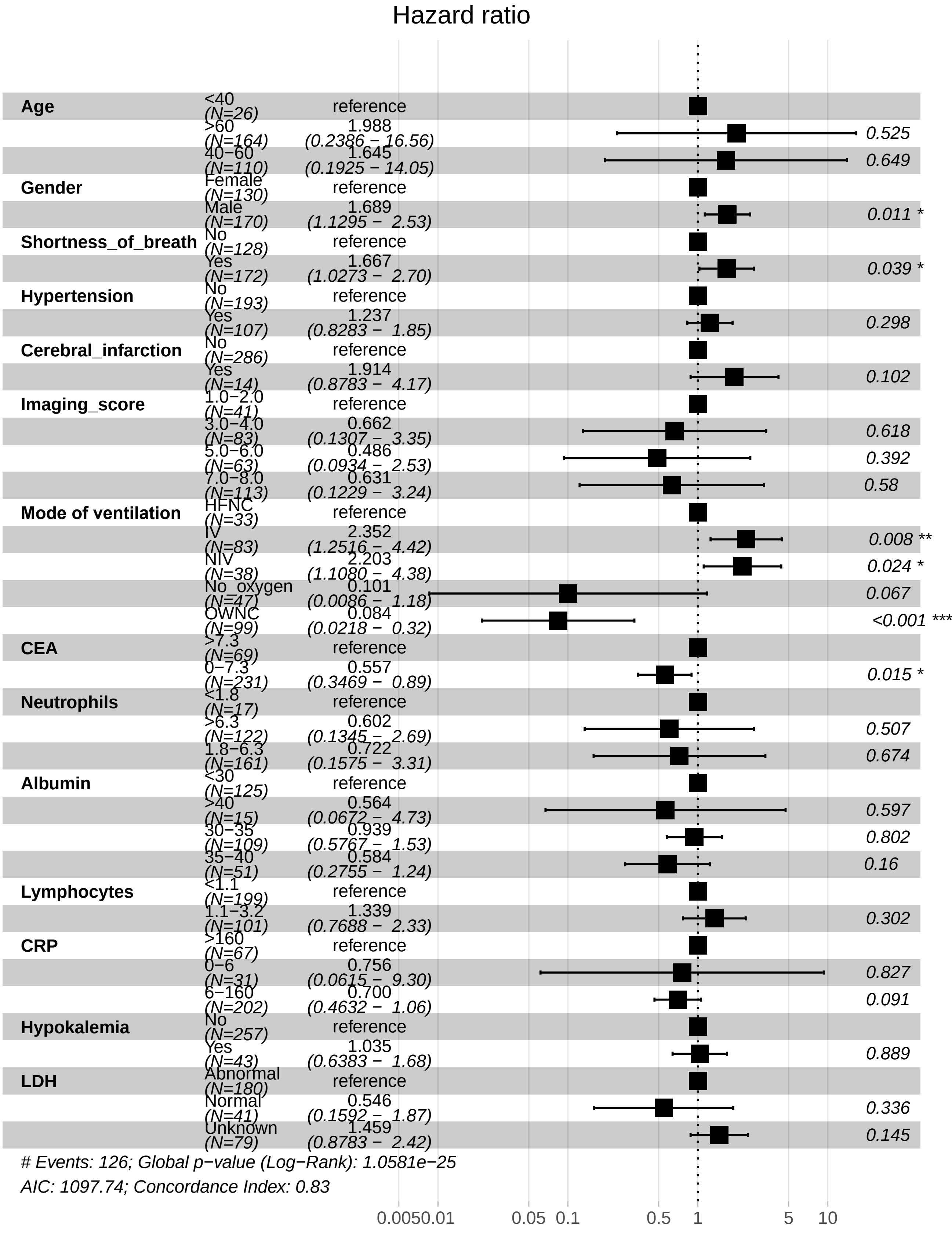

Hazard ratio
Age <40 (N=26) reference
>60 (N=164) 1.988 (0.2386 − 16.56) 0.525
40−60 (N=110) 1.645 (0.1925 − 14.05) 0.649
Gender Female (N=130) reference
Male (N=170) 1.689 (1.1295 − 2.53) 0.011 *
Shortness_of_breath No (N=128) reference
Yes (N=172) 1.667 (1.0273 − 2.70) 0.039 *
Hypertension No (N=193) reference
Yes (N=107) 1.237 (0.8283 − 1.85) 0.298
Cerebral_infarction No (N=286) reference
Yes (N=14) 1.914 (0.8783 − 4.17) 0.102
Imaging_score 1.0−2.0 (N=41) reference
3.0−4.0 (N=83) 0.662 (0.1307 − 3.35) 0.618
5.0−6.0 (N=63) 0.486 (0.0934 − 2.53) 0.392
7.0−8.0 (N=113) 0.631 (0.1229 − 3.24) 0.58
Mode of ventilation HFNC (N=33) reference
IV (N=83) 2.352 (1.2516 − 4.42) 0.008 **
NIV (N=38) 2.203 (1.1080 − 4.38) 0.024 *
No_oxygen (N=47) 0.101 (0.0086 − 1.18) 0.067
OWNC (N=99) 0.084 (0.0218 − 0.32) <0.001 ***
CEA >7.3 (N=69) reference
0−7.3 (N=231) 0.557 (0.3469 − 0.89) 0.015 *
Neutrophils <1.8 (N=17) reference
>6.3 (N=122) 0.602 (0.1345 − 2.69) 0.507
1.8−6.3 (N=161) 0.722 (0.1575 − 3.31) 0.674
Albumin <30 (N=125) reference
>40 (N=15) 0.564 (0.0672 − 4.73) 0.597
30−35 (N=109) 0.939 (0.5767 − 1.53) 0.802
35−40 (N=51) 0.584 (0.2755 − 1.24) 0.16
Lymphocytes <1.1 (N=199) reference
1.1−3.2 (N=101) 1.339 (0.7688 − 2.33) 0.302
CRP >160 (N=67) reference
0−6 (N=31) 0.756 (0.0615 − 9.30) 0.827
6−160 (N=202) 0.700 (0.4632 − 1.06) 0.091
Hypokalemia No (N=257) reference
Yes (N=43) 1.035 (0.6383 − 1.68) 0.889
LDH Abnormal (N=180) reference
Normal (N=41) 0.546 (0.1592 − 1.87) 0.336
Unknown (N=79) 1.459 (0.8783 − 2.42) 0.145
Events: 126; Global p−value (Log−Rank): 1.0581e−25
AIC: 1097.74; Concordance Index: 0.83
0.005 0.01 0.05 0.1 0.5 1 5 10

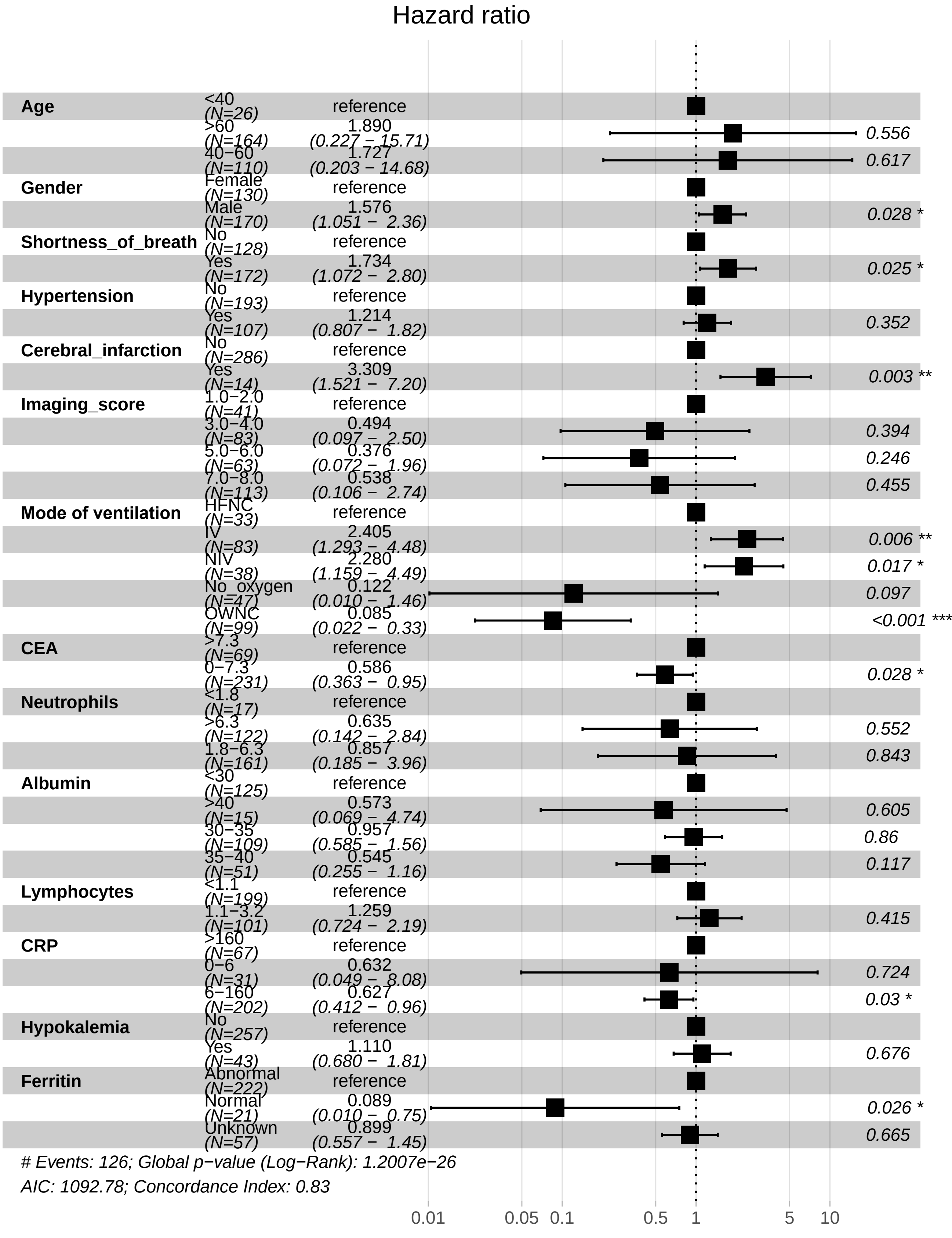

Hazard ratio
Age
<40 (N=26) reference
>60 (N=164) 1.890 (0.227 − 15.71) 0.556
40−60 (N=110) 1.727 (0.203 − 14.68) 0.617
Gender
Female (N=130) reference
Male (N=170) 1.576 (1.051 − 2.36) 0.028 *
Shortness_of_breath
No (N=128) reference
Yes (N=172) 1.734 (1.072 − 2.80) 0.025 *
Hypertension
No (N=193) reference
Yes (N=107) 1.214 (0.807 − 1.82) 0.352
Cerebral_infarction
No (N=286) reference
Yes (N=14) 3.309 (1.521 − 7.20) 0.003 **
Imaging_score
1.0−2.0 (N=41) reference
3.0−4.0 (N=83) 0.494 (0.097 − 2.50) 0.394
5.0−6.0 (N=63) 0.376 (0.072 − 1.96) 0.246
7.0−8.0 (N=113) 0.538 (0.106 − 2.74) 0.455
Mode of ventilation
HFNC (N=33) reference
IV (N=83) 2.405 (1.293 − 4.48) 0.006 **
NIV (N=38) 2.280 (1.159 − 4.49) 0.017 *
No_oxygen (N=47) 0.122 (0.010 − 1.46) 0.097
OWNC (N=99) 0.085 (0.022 − 0.33) <0.001 ***
CEA
>7.3 (N=69) reference
0−7.3 (N=231) 0.586 (0.363 − 0.95) 0.028 *
Neutrophils
<1.8 (N=17) reference
>6.3 (N=122) 0.635 (0.142 − 2.84) 0.552
1.8−6.3 (N=161) 0.857 (0.185 − 3.96) 0.843
Albumin
<30 (N=125) reference
>40 (N=15) 0.573 (0.069 − 4.74) 0.605
30−35 (N=109) 0.957 (0.585 − 1.56) 0.86
35−40 (N=51) 0.545 (0.255 − 1.16) 0.117
Lymphocytes
<1.1 (N=199) reference
1.1−3.2 (N=101) 1.259 (0.724 − 2.19) 0.415
CRP
>160 (N=67) reference
0−6 (N=31) 0.632 (0.049 − 8.08) 0.724
6−160 (N=202) 0.627 (0.412 − 0.96) 0.03 *
Hypokalemia
No (N=257) reference
Yes (N=43) 1.110 (0.680 − 1.81) 0.676
Ferritin
Abnormal (N=222) reference
Normal (N=21) 0.089 (0.010 − 0.75) 0.026 *
Unknown (N=57) 0.899 (0.557 − 1.45) 0.665
Events: 126; Global p−value (Log−Rank): 1.2007e−26
AIC: 1092.78; Concordance Index: 0.83
0.01 0.05 0.1 0.5 1 5 10

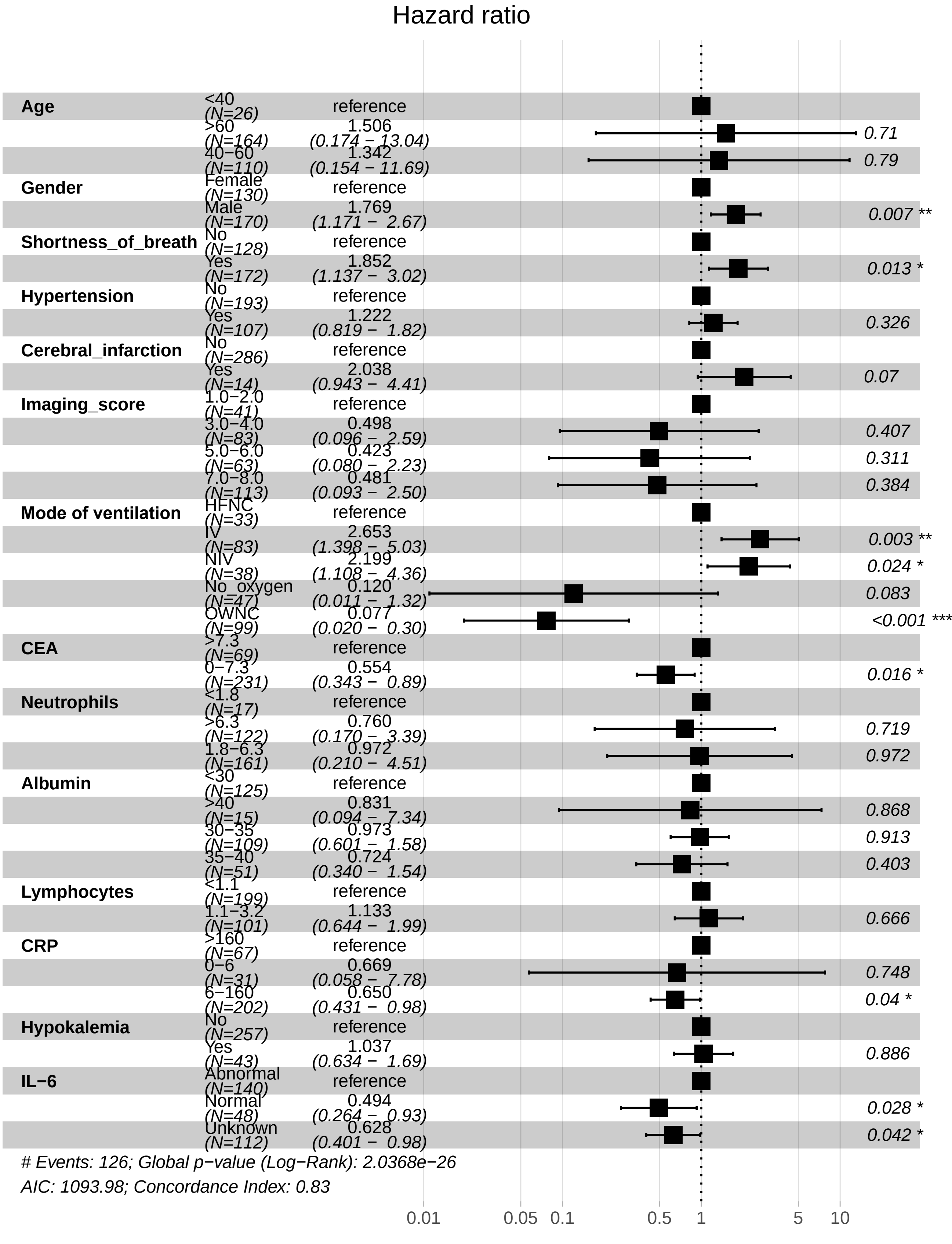
Hazard ratio
Age
<40 (N=26) reference
>60 (N=164) 1.506 (0.174 − 13.04) 0.71
40−60 (N=110) 1.342 (0.154 − 11.69) 0.79
Gender
Female (N=130) reference
Male (N=170) 1.769 (1.171 − 2.67) 0.007 **
Shortness_of_breath
No (N=128) reference
Yes (N=172) 1.852 (1.137 − 3.02) 0.013 *
Hypertension
No (N=193) reference
Yes (N=107) 1.222 (0.819 − 1.82) 0.326
Cerebral_infarction
No (N=286) reference
Yes (N=14) 2.038 (0.943 − 4.41) 0.07
Imaging_score
1.0−2.0 (N=41) reference
3.0−4.0 (N=83) 0.498 (0.096 − 2.59) 0.407
5.0−6.0 (N=63) 0.423 (0.080 − 2.23) 0.311
7.0−8.0 (N=113) 0.481 (0.093 − 2.50) 0.384
Mode of ventilation
HFNC (N=33) reference
IV (N=83) 2.653 (1.398 − 5.03) 0.003 **
NIV (N=38) 2.199 (1.108 − 4.36) 0.024 *
No_oxygen (N=47) 0.120 (0.011 − 1.32) 0.083
OWNC (N=99) 0.077 (0.020 − 0.30) <0.001 ***
CEA
>7.3 (N=69) reference
0−7.3 (N=231) 0.554 (0.343 − 0.89) 0.016 *
Neutrophils
<1.8 (N=17) reference
>6.3 (N=122) 0.760 (0.170 − 3.39) 0.719
1.8−6.3 (N=161) 0.972 (0.210 − 4.51) 0.972
Albumin
<30 (N=125) reference
>40 (N=15) 0.831 (0.094 − 7.34) 0.868
30−35 (N=109) 0.973 (0.601 − 1.58) 0.913
35−40 (N=51) 0.724 (0.340 − 1.54) 0.403
Lymphocytes
<1.1 (N=199) reference
1.1−3.2 (N=101) 1.133 (0.644 − 1.99) 0.666
CRP
>160 (N=67) reference
0−6 (N=31) 0.669 (0.058 − 7.78) 0.748
6−160 (N=202) 0.650 (0.431 − 0.98) 0.04 *
Hypokalemia
No (N=257) reference
Yes (N=43) 1.037 (0.634 − 1.69) 0.886
IL−6
Abnormal (N=140) reference
Normal (N=48) 0.494 (0.264 − 0.93) 0.028 *
Unknown (N=112) 0.628 (0.401 − 0.98) 0.042 *
Events: 126; Global p−value (Log−Rank): 2.0368e−26
AIC: 1093.98; Concordance Index: 0.83
0.01
0.05
0.1
0.5
1
5
10

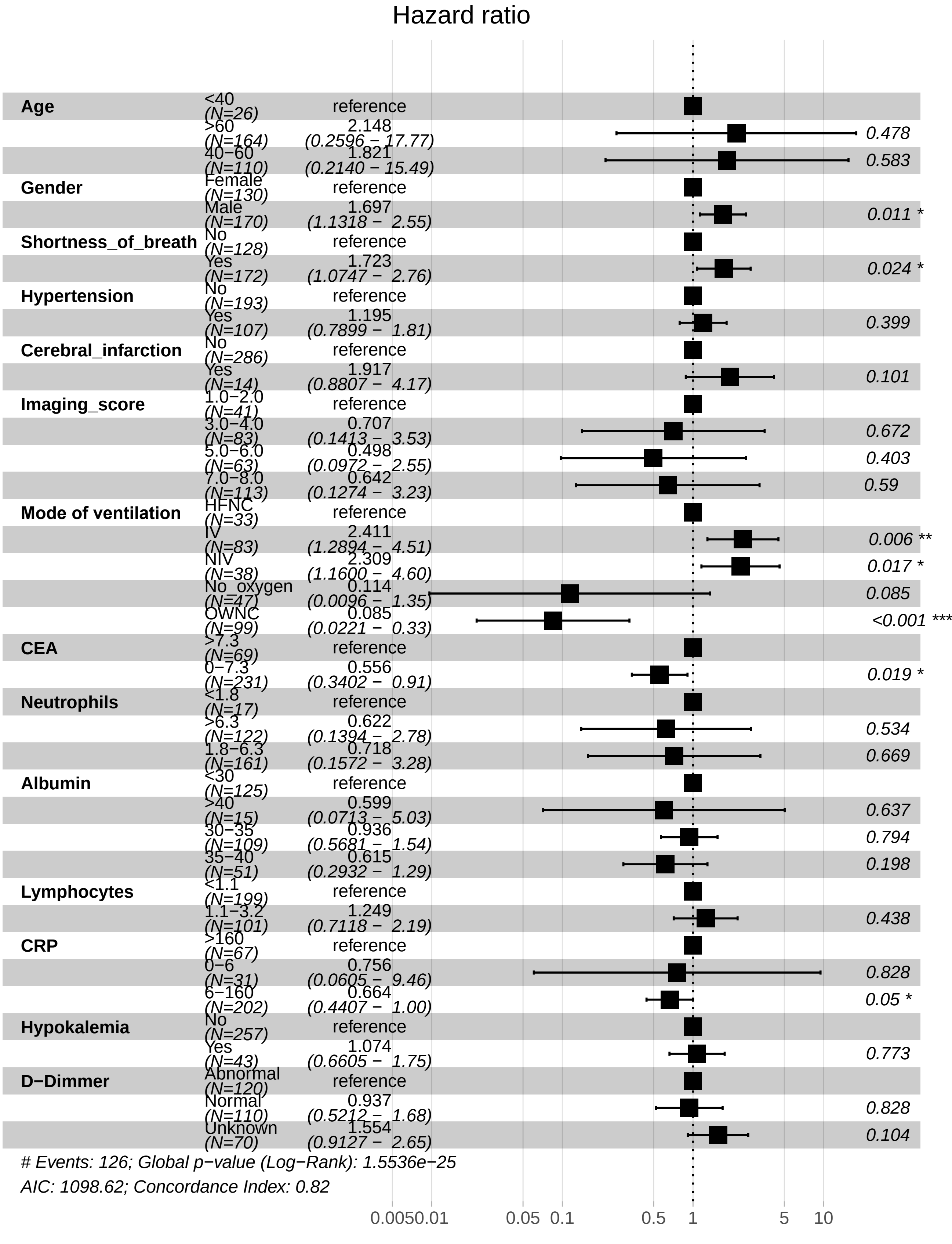
Hazard ratio
Age
<40 (N=26) reference
>60 (N=164) 2.148 (0.2596 − 17.77) 0.478
40−60 (N=110) 1.821 (0.2140 − 15.49) 0.583
Gender
Female (N=130) reference
Male (N=170) 1.697 (1.1318 − 2.55) 0.011 *
Shortness_of_breath
No (N=128) reference
Yes (N=172) 1.723 (1.0747 − 2.76) 0.024 *
Hypertension
No (N=193) reference
Yes (N=107) 1.195 (0.7899 − 1.81) 0.399
Cerebral_infarction
No (N=286) reference
Yes (N=14) 1.917 (0.8807 − 4.17) 0.101
Imaging_score
1.0−2.0 (N=41) reference
3.0−4.0 (N=83) 0.707 (0.1413 − 3.53) 0.672
5.0−6.0 (N=63) 0.498 (0.0972 − 2.55) 0.403
7.0−8.0 (N=113) 0.642 (0.1274 − 3.23) 0.59
Mode of ventilation
HFNC (N=33) reference
IV (N=83) 2.411 (1.2894 − 4.51) 0.006 **
NIV (N=38) 2.309 (1.1600 − 4.60) 0.017 *
No_oxygen (N=47) 0.114 (0.0096 − 1.35) 0.085
OWNC (N=99) 0.085 (0.0221 − 0.33) <0.001 ***
CEA
>7.3 (N=69) reference
0−7.3 (N=231) 0.556 (0.3402 − 0.91) 0.019 *
Neutrophils
<1.8 (N=17) reference
>6.3 (N=122) 0.622 (0.1394 − 2.78) 0.534
1.8−6.3 (N=161) 0.718 (0.1572 − 3.28) 0.669
Albumin
<30 (N=125) reference
>40 (N=15) 0.599 (0.0713 − 5.03) 0.637
30−35 (N=109) 0.936 (0.5681 − 1.54) 0.794
35−40 (N=51) 0.615 (0.2932 − 1.29) 0.198
Lymphocytes
<1.1 (N=199) reference
1.1−3.2 (N=101) 1.249 (0.7118 − 2.19) 0.438
CRP
>160 (N=67) reference
0−6 (N=31) 0.756 (0.0605 − 9.46) 0.828
6−160 (N=202) 0.664 (0.4407 − 1.00) 0.05 *
Hypokalemia
No (N=257) reference
Yes (N=43) 1.074 (0.6605 − 1.75) 0.773
D−Dimmer
Abnormal (N=120) reference
Normal (N=110) 0.937 (0.5212 − 1.68) 0.828
Unknown (N=70) 1.554 (0.9127 − 2.65) 0.104
Events: 126; Global p−value (Log−Rank): 1.5536e−25
AIC: 1098.62; Concordance Index: 0.82
0.005 0.01 0.05 0.1 0.5 1 5 10

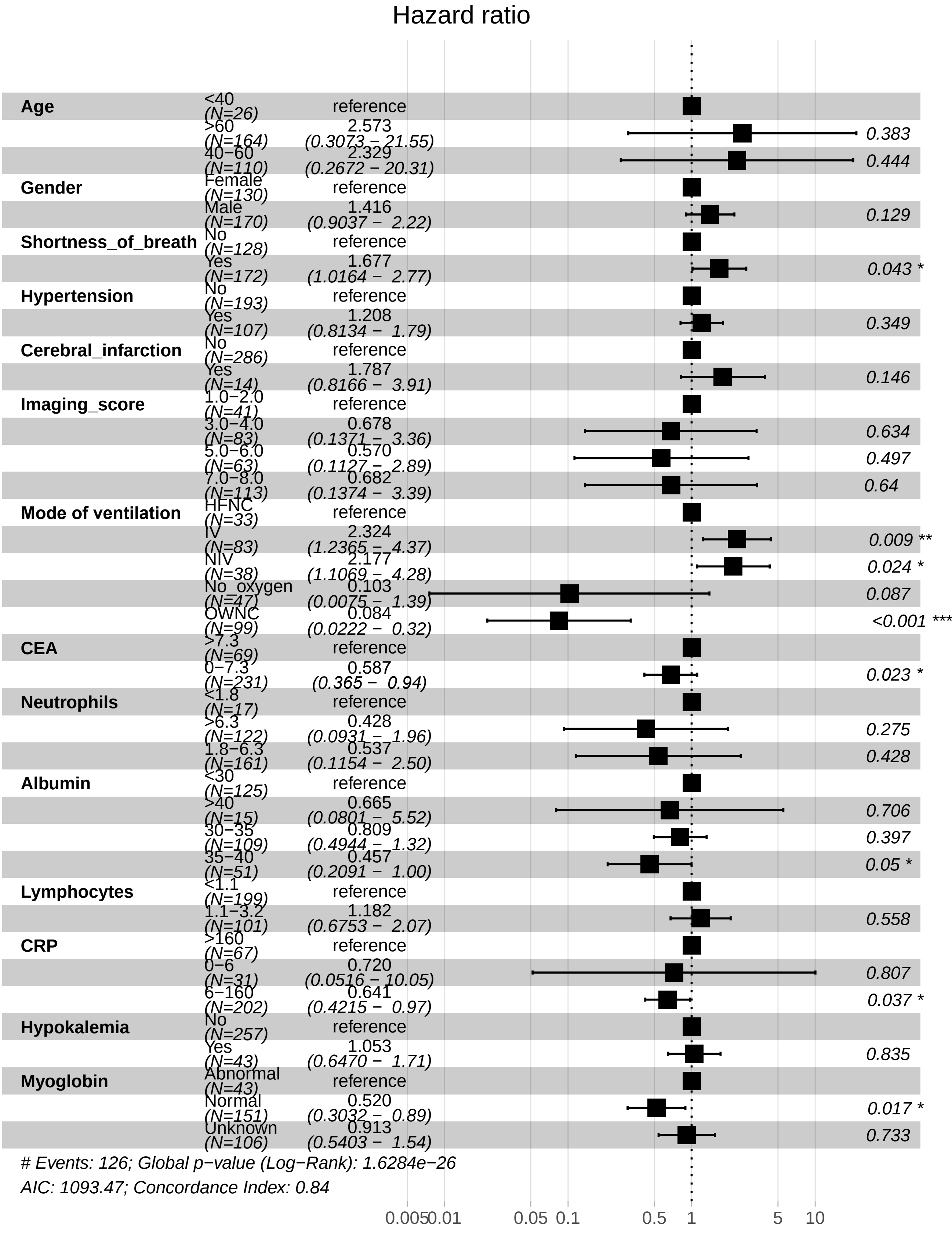

Hazard ratio
Age
<40 (N=26) reference
>60 (N=164) 2.573 (0.3073 − 21.55) 0.383
40−60 (N=110) 2.329 (0.2672 − 20.31) 0.444
Gender
Female (N=130) reference
Male (N=170) 1.416 (0.9037 − 2.22) 0.129
Shortness_of_breath
No (N=128) reference
Yes (N=172) 1.677 (1.0164 − 2.77) 0.043 *
Hypertension
No (N=193) reference
Yes (N=107) 1.208 (0.8134 − 1.79) 0.349
Cerebral_infarction
No (N=286) reference
Yes (N=14) 1.787 (0.8166 − 3.91) 0.146
Imaging_score
1.0−2.0 (N=41) reference
3.0−4.0 (N=83) 0.678 (0.1371 − 3.36) 0.634
5.0−6.0 (N=63) 0.570 (0.1127 − 2.89) 0.497
7.0−8.0 (N=113) 0.682 (0.1374 − 3.39) 0.64
Mode of ventilation
HFNC (N=33) reference
IV (N=83) 2.324 (1.2365 − 4.37) 0.009 **
NIV (N=38) 2.177 (1.1069 − 4.28) 0.024 *
No_oxygen (N=47) 0.103 (0.0075 − 1.39) 0.087
OWNC (N=99) 0.084 (0.0222 − 0.32) <0.001 ***
CEA
>7.3 (N=69) reference
0−7.3 (N=231) 0.587 (0.365 − 0.94) 0.023 *
Neutrophils
<1.8 (N=17) reference
>6.3 (N=122) 0.428 (0.0931 − 1.96) 0.275
1.8−6.3 (N=161) 0.537 (0.1154 − 2.50) 0.428
Albumin
<30 (N=125) reference
>40 (N=15) 0.665 (0.0801 − 5.52) 0.706
30−35 (N=109) 0.809 (0.4944 − 1.32) 0.397
35−40 (N=51) 0.457 (0.2091 − 1.00) 0.05 *
Lymphocytes
<1.1 (N=199) reference
1.1−3.2 (N=101) 1.182 (0.6753 − 2.07) 0.558
CRP
>160 (N=67) reference
0−6 (N=31) 0.720 (0.0516 − 10.05) 0.807
6−160 (N=202) 0.641 (0.4215 − 0.97) 0.037 *
Hypokalemia
No (N=257) reference
Yes (N=43) 1.053 (0.6470 − 1.71) 0.835
Myoglobin
Abnormal (N=43) reference
Normal (N=151) 0.520 (0.3032 − 0.89) 0.017 *
Unknown (N=106) 0.913 (0.5403 − 1.54) 0.733
Events: 126; Global p−value (Log−Rank): 1.6284e−26
AIC: 1093.47; Concordance Index: 0.84
0.005 0.01 0.05 0.1 0.5 1 5 10

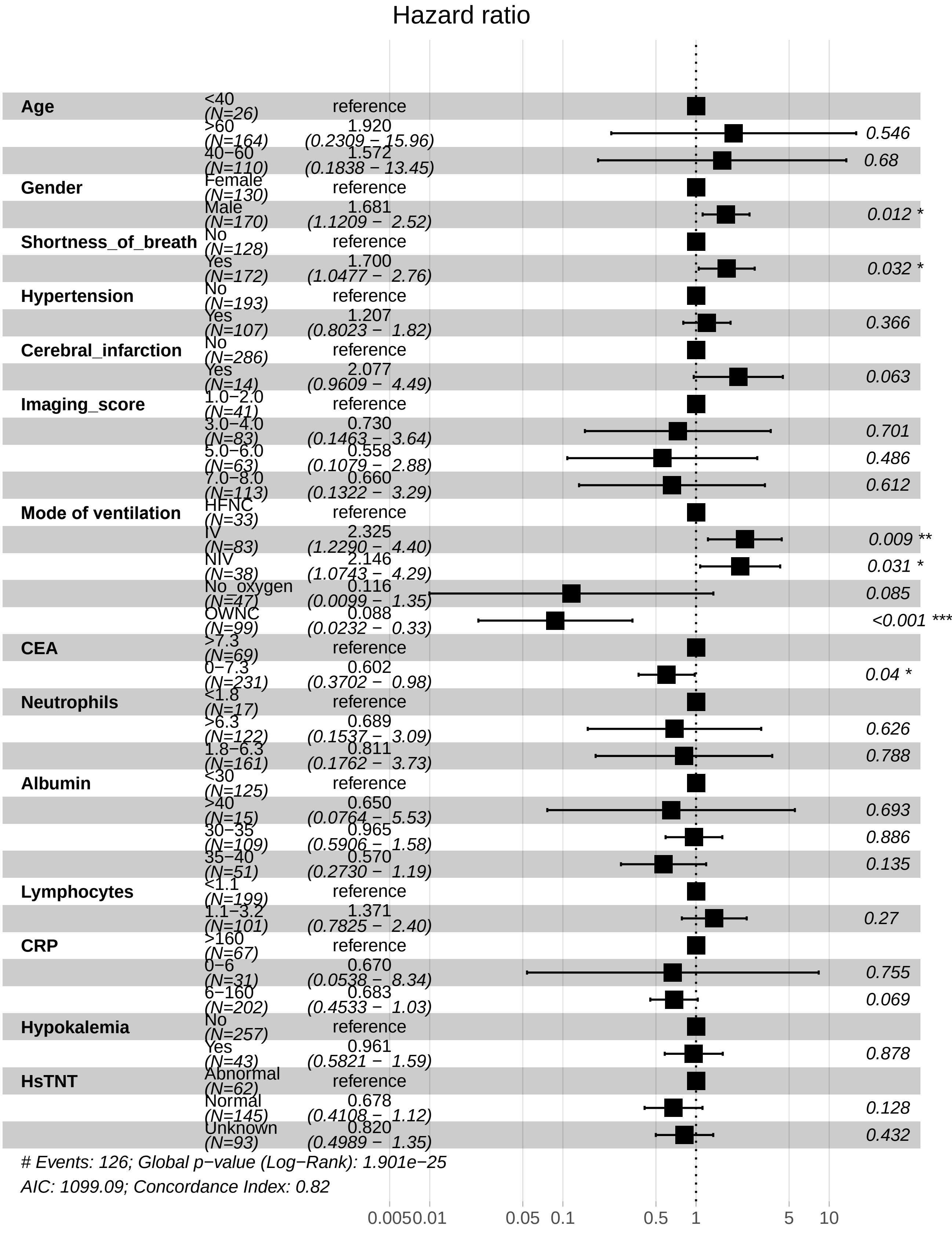
Hazard ratio
Age
<40 (N=26) reference
>60 (N=164) 1.920 (0.2309 − 15.96) 0.546
40−60 (N=110) 1.572 (0.1838 − 13.45) 0.68
Gender
Female (N=130) reference
Male (N=170) 1.681 (1.1209 − 2.52) 0.012 *
Shortness_of_breath
No (N=128) reference
Yes (N=172) 1.700 (1.0477 − 2.76) 0.032 *
Hypertension
No (N=193) reference
Yes (N=107) 1.207 (0.8023 − 1.82) 0.366
Cerebral_infarction
No (N=286) reference
Yes (N=14) 2.077 (0.9609 − 4.49) 0.063
Imaging_score
1.0−2.0 (N=41) reference
3.0−4.0 (N=83) 0.730 (0.1463 − 3.64) 0.701
5.0−6.0 (N=63) 0.558 (0.1079 − 2.88) 0.486
7.0−8.0 (N=113) 0.660 (0.1322 − 3.29) 0.612
Mode of ventilation
HFNC (N=33) reference
IV (N=83) 2.325 (1.2290 − 4.40) 0.009 **
NIV (N=38) 2.146 (1.0743 − 4.29) 0.031 *
No_oxygen (N=47) 0.116 (0.0099 − 1.35) 0.085
OWNC (N=99) 0.088 (0.0232 − 0.33) <0.001 ***
CEA
>7.3 (N=69) reference
0−7.3 (N=231) 0.602 (0.3702 − 0.98) 0.04 *
Neutrophils
<1.8 (N=17) reference
>6.3 (N=122) 0.689 (0.1537 − 3.09) 0.626
1.8−6.3 (N=161) 0.811 (0.1762 − 3.73) 0.788
Albumin
<30 (N=125) reference
>40 (N=15) 0.650 (0.0764 − 5.53) 0.693
30−35 (N=109) 0.965 (0.5906 − 1.58) 0.886
35−40 (N=51) 0.570 (0.2730 − 1.19) 0.135
Lymphocytes
<1.1 (N=199) reference
1.1−3.2 (N=101) 1.371 (0.7825 − 2.40) 0.27
CRP
>160 (N=67) reference
0−6 (N=31) 0.670 (0.0538 − 8.34) 0.755
6−160 (N=202) 0.683 (0.4533 − 1.03) 0.069
Hypokalemia
No (N=257) reference
Yes (N=43) 0.961 (0.5821 − 1.59) 0.878
HsTNT
Abnormal (N=62) reference
Normal (N=145) 0.678 (0.4108 − 1.12) 0.128
Unknown (N=93) 0.820 (0.4989 − 1.35) 0.432
Events: 126; Global p−value (Log−Rank): 1.901e−25
AIC: 1099.09; Concordance Index: 0.82
0.005 0.01 0.05 0.1 0.5 1 5 10
