# Supplementary material for: The predicting roles of carcinoembryonic antigen and its underlying mechanism in the progression of coronavirus disease 2019
Source: Crit Care. 2021 Jul 3;25:234. doi: 10.1186/s13054-021-03661-y (PMC8254455; doi:10.1186/s13054-021-03661-y)
Supplement: Supplementary file 4 — Additional file 4: Supplementary material 3: Multivariate Cox regression model including smoking information (keep or remove missing values). As smokers or patients with specific malignancies could have increased CEA levels without any correlation with COVID-19, all patients with primary malignancy were excluded from the study. Furthermore, in order to identify the association between CEA levels and smoking, two subgroup Cox proportional hazard regression models including smoking status (keep or remove missing values) were constructed, suggesting that the CEA level (HR 0.547; 95% CI 0.318 to 0.940; P = 0.037) (remove missing values) (HR 0.620; 95% CI 0.384 to 0.990; P = 0.048) (keep missing values) was still an independent prognostic indicator for COVID-19 patients. [file 13054_2021_3661_MOESM4_ESM.pdf]

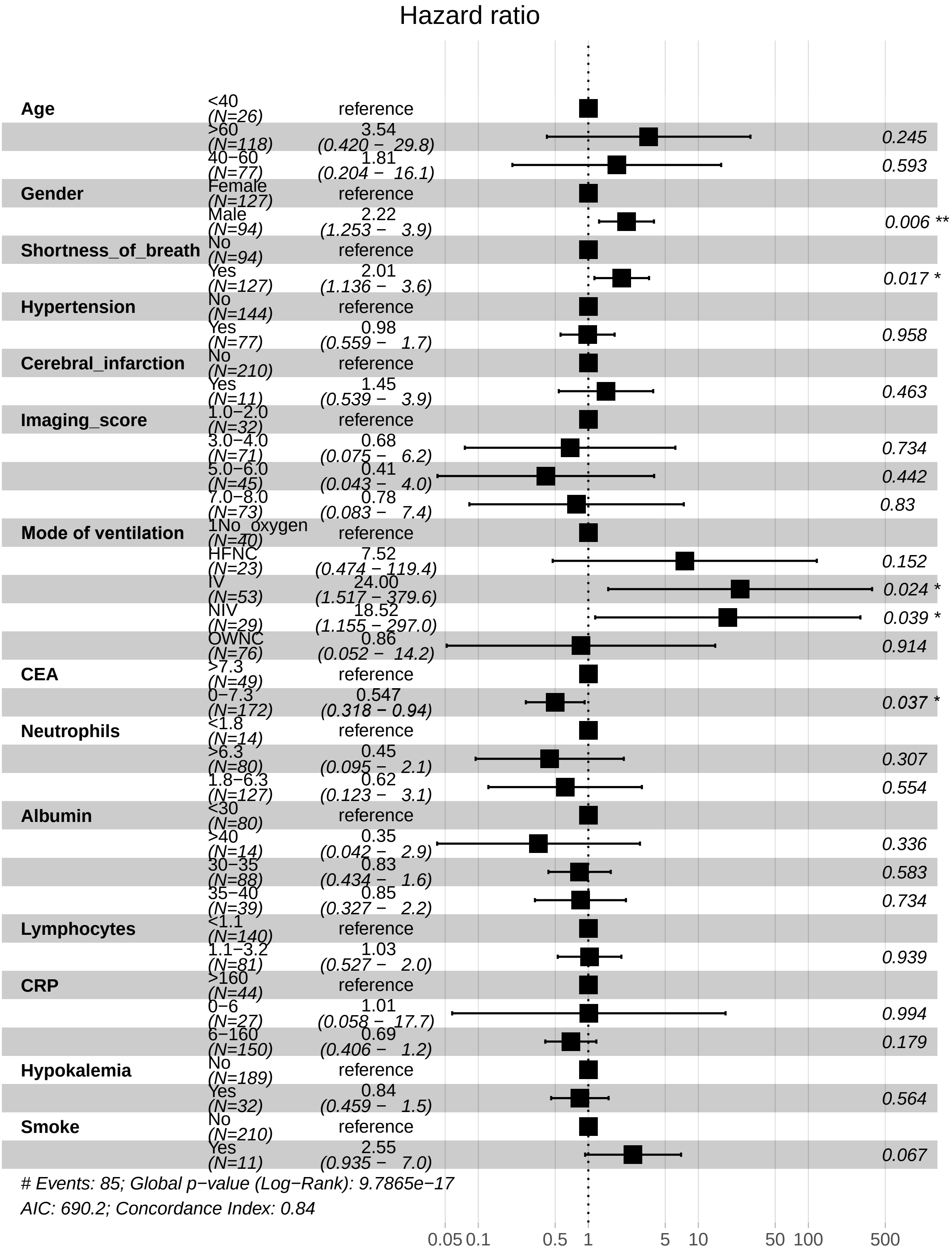
Hazard ratio
Age
<40 (N=26) reference
>60 (N=118) 3.54 (0.420 − 29.8) 0.245
40−60 (N=77) 1.81 (0.204 − 16.1) 0.593
Gender
Female (N=127) reference
Male (N=94) 2.22 (1.253 − 3.9) 0.006 **
Shortness_of_breath
No (N=94) reference
Yes (N=127) 2.01 (1.136 − 3.6) 0.017 *
Hypertension
No (N=144) reference
Yes (N=77) 0.98 (0.559 − 1.7) 0.958
Cerebral_infarction
No (N=210) reference
Yes (N=11) 1.45 (0.539 − 3.9) 0.463
Imaging_score
1.0−2.0 (N=32) reference
3.0−4.0 (N=71) 0.68 (0.075 − 6.2) 0.734
5.0−6.0 (N=45) 0.41 (0.043 − 4.0) 0.442
7.0−8.0 (N=73) 0.78 (0.083 − 7.4) 0.83
Mode of ventilation
1No_oxygen (N=40) reference
HFNC (N=23) 7.52 (0.474 − 119.4) 0.152
IV (N=53) 24.00 (1.517 − 379.6) 0.024 *
NIV (N=29) 18.52 (1.155 − 297.0) 0.039 *
OWNC (N=76) 0.86 (0.052 − 14.2) 0.914
CEA
>7.3 (N=49) reference
0−7.3 (N=172) 0.547 (0.318 − 0.94) 0.037 *
Neutrophils
<1.8 (N=14) reference
>6.3 (N=80) 0.45 (0.095 − 2.1) 0.307
1.8−6.3 (N=127) 0.62 (0.123 − 3.1) 0.554
Albumin
<30 (N=80) reference
>40 (N=14) 0.35 (0.042 − 2.9) 0.336
30−35 (N=88) 0.83 (0.434 − 1.6) 0.583
35−40 (N=39) 0.85 (0.327 − 2.2) 0.734
Lymphocytes
<1.1 (N=140) reference
1.1−3.2 (N=81) 1.03 (0.527 − 2.0) 0.939
CRP
>160 (N=44) reference
0−6 (N=27) 1.01 (0.058 − 17.7) 0.994
6−160 (N=150) 0.69 (0.406 − 1.2) 0.179
Hypokalemia
No (N=189) reference
Yes (N=32) 0.84 (0.459 − 1.5) 0.564
Smoke
No (N=210) reference
Yes (N=11) 2.55 (0.935 − 7.0) 0.067
Events: 85; Global p−value (Log−Rank): 9.7865e−17
AIC: 690.2; Concordance Index: 0.84
0.05 0.1 0.5 1 5 10 50 100 500

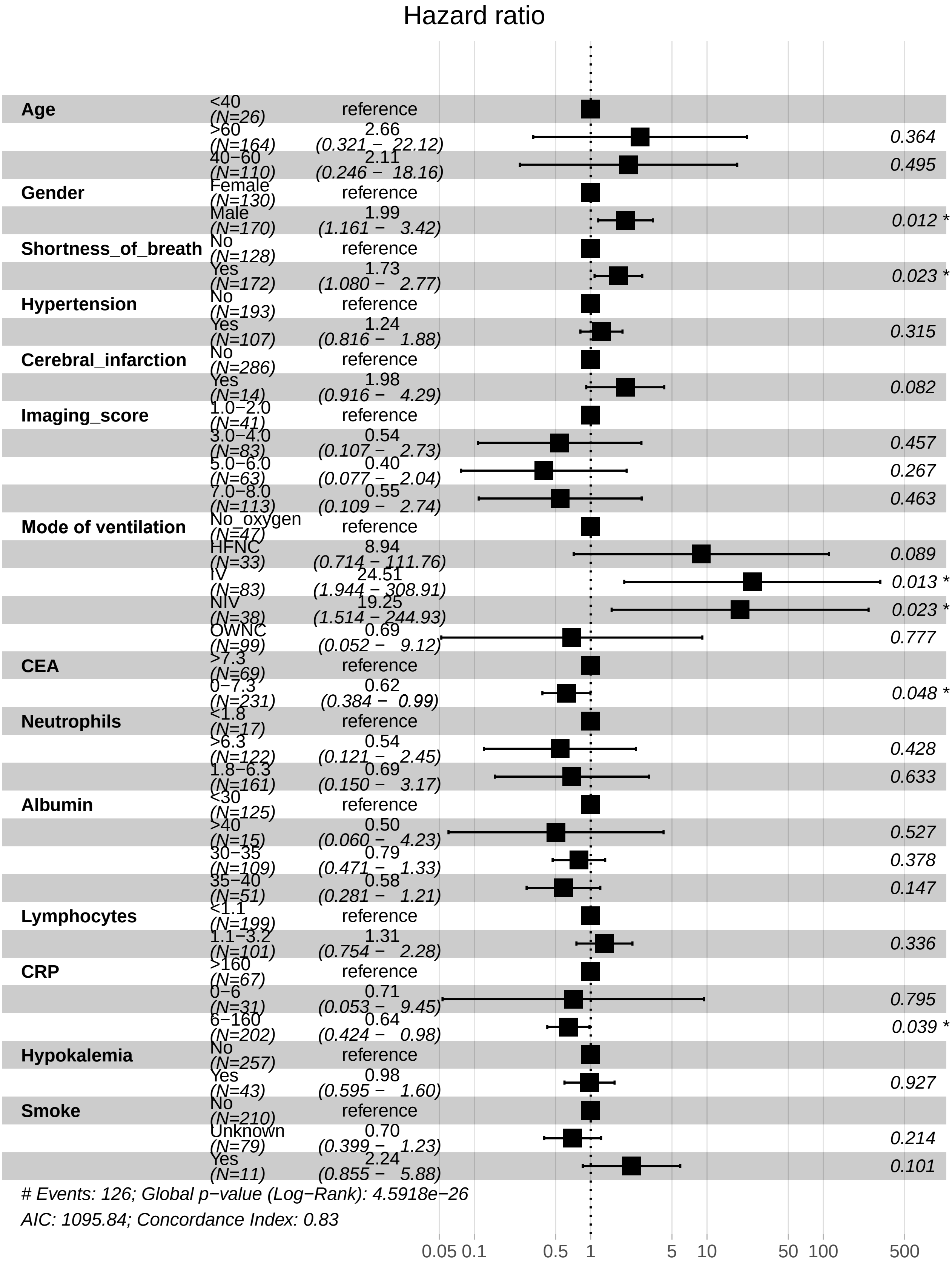
Hazard ratio
Age
<40 (N=26) reference
>60 (N=164) 2.66 (0.321 − 22.12) 0.364
40−60 (N=110) 2.11 (0.246 − 18.16) 0.495
Gender
Female (N=130) reference
Male (N=170) 1.99 (1.161 − 3.42) 0.012 *
Shortness_of_breath
No (N=128) reference
Yes (N=172) 1.73 (1.080 − 2.77) 0.023 *
Hypertension
No (N=193) reference
Yes (N=107) 1.24 (0.816 − 1.88) 0.315
Cerebral_infarction
No (N=286) reference
Yes (N=14) 1.98 (0.916 − 4.29) 0.082
Imaging_score
1.0−2.0 (N=41) reference
3.0−4.0 (N=83) 0.54 (0.107 − 2.73) 0.457
5.0−6.0 (N=63) 0.40 (0.077 − 2.04) 0.267
7.0−8.0 (N=113) 0.55 (0.109 − 2.74) 0.463
Mode of ventilation
No_oxygen (N=47) reference
HFNC (N=33) 8.94 (0.714 − 111.76) 0.089
IV (N=83) 24.51 (1.944 − 308.91) 0.013 *
NIV (N=38) 19.25 (1.514 − 244.93) 0.023 *
OWNC (N=99) 0.69 (0.052 − 9.12) 0.777
CEA
>7.3 (N=69) reference
0−7.3 (N=231) 0.62 (0.384 − 0.99) 0.048 *
Neutrophils
<1.8 (N=17) reference
>6.3 (N=122) 0.54 (0.121 − 2.45) 0.428
1.8−6.3 (N=161) 0.69 (0.150 − 3.17) 0.633
Albumin
<30 (N=125) reference
>40 (N=15) 0.50 (0.060 − 4.23) 0.527
30−35 (N=109) 0.79 (0.471 − 1.33) 0.378
35−40 (N=51) 0.58 (0.281 − 1.21) 0.147
Lymphocytes
<1.1 (N=199) reference
1.1−3.2 (N=101) 1.31 (0.754 − 2.28) 0.336
CRP
>160 (N=67) reference
0−6 (N=31) 0.71 (0.053 − 9.45) 0.795
6−160 (N=202) 0.64 (0.424 − 0.98) 0.039 *
Hypokalemia
No (N=257) reference
Yes (N=43) 0.98 (0.595 − 1.60) 0.927
Smoke
No (N=210) reference
Unknown (N=79) 0.70 (0.399 − 1.23) 0.214
Yes (N=11) 2.24 (0.855 − 5.88) 0.101
Events: 126; Global p−value (Log−Rank): 4.5918e−26
AIC: 1095.84; Concordance Index: 0.83
0.05 0.1 0.5 1 5 10 50 100 500
