# Supplementary figures and images for: The predicting roles of carcinoembryonic antigen and its underlying mechanism in the progression of coronavirus disease 2019
Source: Crit Care. 2021 Jul 3;25:234. doi: 10.1186/s13054-021-03661-y (PMC8254455; doi:10.1186/s13054-021-03661-y)

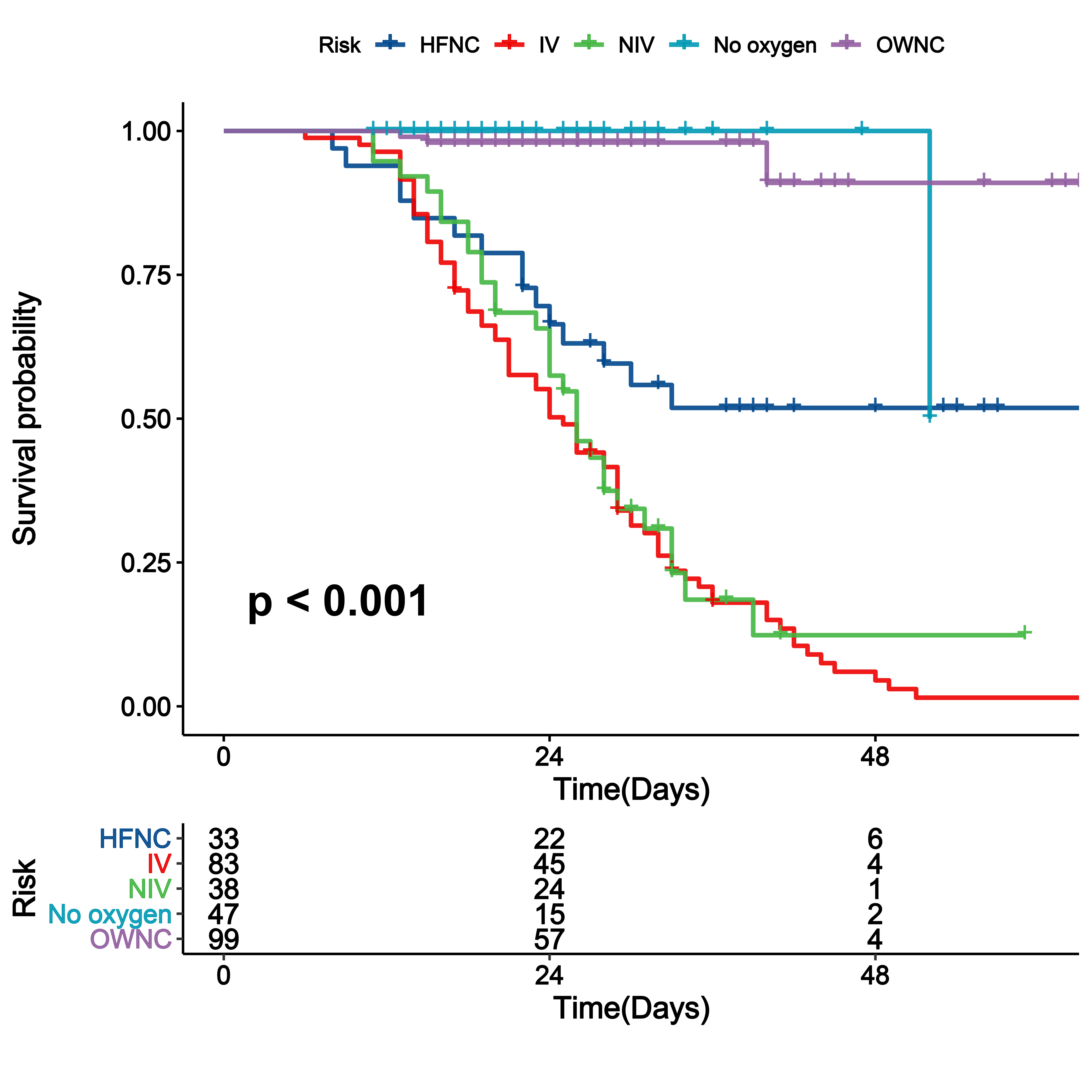

Supplement: Supplementary file 7 — Additional file 7. Figure S3: Kaplan–Meier curve evaluating the prognostic value of the mode of ventilation. To further evaluate the prognostic value of mode of ventilation, the Kaplan–Meier analysis was performed. The results suggested that mode of ventilation was significantly associated with the prognosis of COVID-19 patients (P < 0.001). [file 13054_2021_3661_MOESM7_ESM.jpg]
